# Transcriptomic Response Analysis of *Escherichia coli* to Palladium Stress

**DOI:** 10.3389/fmicb.2021.741836

**Published:** 2021-10-08

**Authors:** Nadeem Joudeh, Athanasios Saragliadis, Christian Schulz, André Voigt, Eivind Almaas, Dirk Linke

**Affiliations:** ^1^Department of Biosciences, University of Oslo, Oslo, Norway; ^2^Department of Biotechnology and Food Science, Norwegian University of Science and Technology (NTNU), Trondheim, Norway

**Keywords:** RNA-seq, heavy metal stress, palladium, precious metals, nanoparticles, oxidative stress, *Escherichia coli*, transcriptional response

## Abstract

Palladium (Pd), due to its unique catalytic properties, is an industrially important heavy metal especially in the form of nanoparticles. It has a wide range of applications from automobile catalytic converters to the pharmaceutical production of morphine. Bacteria have been used to biologically produce Pd nanoparticles as a new environmentally friendly alternative to the currently used energy-intensive and toxic physicochemical methods. Heavy metals, including Pd, are toxic to bacterial cells and cause general and oxidative stress that hinders the use of bacteria to produce Pd nanoparticles efficiently. In this study, we show in detail the Pd stress-related effects on *E. coli*. Pd stress effects were measured as changes in the transcriptome through RNA-Seq after 10 min of exposure to 100 μM sodium tetrachloropalladate (II). We found that 709 out of 3,898 genes were differentially expressed, with 58% of them being up-regulated and 42% of them being down-regulated. Pd was found to induce several common heavy metal stress-related effects but interestingly, Pd causes unique effects too. Our data suggests that Pd disrupts the homeostasis of Fe, Zn, and Cu cellular pools. In addition, the expression of inorganic ion transporters in *E. coli* was found to be massively modulated due to Pd intoxication, with 17 out of 31 systems being affected. Moreover, the expression of several carbohydrate, amino acid, and nucleotide transport and metabolism genes was vastly changed. These results bring us one step closer to the generation of genetically engineered *E. coli* strains with enhanced capabilities for Pd nanoparticles synthesis.

## Introduction

The International Union of Pure and Applied Chemistry (IUPAC) reported in 2002 that there were more than 30 different definitions used in literature for a heavy metal (Duffus, 2002; Pourret, 2018). Nevertheless, density in most cases is considered to be the defining criterion, and heavy metals are commonly defined by a density higher than 5 g/cm^3^ (Lozet and Mathieu, 1993; Nies, 1999; Järup, 2003). Most heavy metals are transition metals with partially filled d orbitals. These d orbitals provide the cations of heavy metals with the ability to form complex compounds which may be redox-active (Nies, 1999). Hence, heavy-metal cations play an important role as trace elements in biological systems.

From a physiological point of view, heavy metals are placed in two categories: (1) essential ones, also known as trace elements, and (2) toxic heavy metals. The essential heavy metals [Co, Cu, Fe, Mn, Mo, Ni, V, W, and Zn (Nies and Silver, 2007)] are required in smaller amounts than the bulk metals (Na, K, Mg, and Ca). The essential heavy metals have to be acquired by bacteria from their surrounding environments as inorganic ions (Nies and Silver, 2007). These cations are required for functions that include stabilizing biological molecules (Nies and Silver, 2007), electron transfer and redox processes (Bruins et al., 2000), and as components of metalloenzymes, which account for approximately 30% of all enzymes in bacterial cells (Wackett et al., 2004). The toxic heavy metals are elements that have no known beneficial roles and can be damaging to the cell if taken up (Nies and Silver, 2007). The list of toxic heavy metals includes e.g., Ag, Au, Bi, Cd, Cr, Hg, In, Ir, Pb, Pd, Pt, Sn, and Tl (Nies, 1999; Nies and Silver, 2007).

Both excessive levels of essential heavy-metal ions and the presence of toxic heavy-metal ions cause cellular stress (Nies and Silver, 2007). Unlike organic molecules, heavy metals cannot be broken down by enzymatic reactions, and due to the wide range of toxic effects of some heavy metals, strategies for dealing with those toxic heavy metals are limited (Nies and Silver, 2007). Those strategies include (1) the extracellular detoxification and sequestration of the metal, (2) the prevention of the metal from entering the cell by reducing cell permeability, (3) the active transport of the metal out of the cell, (4) the intracellular sequestration of the metal by binding to proteins, and (5) the enzymatic alteration of the metal to a less toxic form (Rouch et al., 1995).

Bacterial cellular responses to stress caused by metal ions can be measured as changes in the transcription of genes involved in the detoxification processes or metal ion homeostasis. Bacterial transcriptional responses to excessive levels of essential heavy metals have been intensively studied in literature (Kershaw et al., 2005; Lee et al., 2005; Fantino et al., 2010; Gault et al., 2016), but for the toxic heavy metals, this information is often lacking or only exists for key environmental pollutants (Wang and Crowley, 2005; LaVoie and Summers, 2018).

Palladium (Pd), element number 46 in the periodic table, belongs to the platinum group metals and is an industrially important heavy metal due to its unique catalytic properties. It is primarily used as a key component in catalysts for different carbon-carbon coupling reactions such as Heck and Suzuki coupling reactions (Cui et al., 2017; Miller et al., 2017; Biffis et al., 2018). The unique ground-state structure of Pd (4d^10^5s^0^) and the square-planar geometry of Pd(II) complexes (d^8^) give Pd unique properties in C-C bond formation and C-O bond cleavage (Jbara et al., 2017). Moreover, Pd is also known for its uniquely high capacity for hydrogen gas absorption (Gavia and Shon, 2015). Besides its major application in automobile catalytic converters (Zereini and Alt, 2006), it is used in versatile applications in catalysis (Biffis et al., 2018), sensors (Kundu et al., 2015), fuel cells (Feliciano-Ramos et al., 2014), and electronics (Zhang G. et al., 2019). Due to its increased industrial use and limited supply, its price has increased more than 27 times in the last 30 years (3,000$/kg in 1990 to 83,000$/kg in 2021)^1^. Consequently, the recovery of waste Pd has become of prime importance. The current techniques for Pd recovery include costly and non-environmentally friendly processes, such as pyrometallurgy, solvent extraction, chemical treatment and electrochemical recovery (Baxter-Plant et al., 2002). Therefore, alternative eco-friendly processes for Pd recovery, such as biomineralization, are of high interest (Lloyd et al., 2020).

Pd ions can be taken up and reduced to Pd nanoparticles by living organisms, such as plants (Vishnukumar et al., 2017), fungi (Tarver et al., 2019), and microorganisms (De Corte et al., 2012). Microorganisms and especially bacteria, due to their fast growth rate and inexpensive cultivation media, are considered one of the most efficient systems for the reduction of Pd (Matsumoto et al., 2020). The formation of Pd nanoparticles through bioreductive deposition of Pd^2+^ ions was intensively studied in different bacteria (De Corte et al., 2012). This metabolic side process is believed to happen during hydrogen production through anaerobic fermentation in *E. coli* (Deplanche et al., 2010) and other obligate and facultative anaerobes (Hennebel et al., 2011), or in the process of sulfate and metal reduction in sulfate-reducing (Lloyd et al., 1998) and metal-reducing bacteria (De Windt et al., 2005). Due to its advantageous properties and its status as a model organism, *E. coli* has been widely used in the recovery of heavy metals and the reduction of metal ions for nanoparticles synthesis (Du et al., 2007; Gurunathan et al., 2009; Quintelas et al., 2009).

The use of *E. coli* whether for Pd recovery or Pd nanoparticles synthesis requires a deep understanding of the exposure effects that Pd causes to *E. coli*. In addition, the genetic engineering of *E. coli* strains that are capable of performing Pd reduction for different applications, requires information about the genes and pathways that are involved in Pd ion uptake from the surrounding environment, in the reduction of Pd ions to form Pd nanoparticles, and in Pd detoxification processes. In this study, we measured the bacterial transcriptional response to the exposure to sublethal levels of Pd^2+^ ions in *E. coli* K-12 BW25113 strain and compared our results with published transcriptional responses to other heavy metals. Anaerobic conditions were approximated in our experiment since Pd nanoparticles synthesis is typically performed in the absence of oxygen.

## Materials and Methods

### Bacterial Strains and Growth Conditions

The bacterial strain used in this study was *E. coli* K-12 BW25113 (Baba et al., 2006). For each biological replicate, a single colony of bacteria was used to inoculate a 10 ml starter culture in M63 minimal medium [(NH_4_)_2_SO_4_ 2 g/L, KH_2_PO_4_ 13.6 g/L, and FeSO_4_.7H_2_O 0.5 mg/L were mixed together, pH was adjusted to 7 with KOH, the solution was autoclaved and then 1 ml of 1 M sterile MgSO_4_.7H_2_O and sterile glucose to a final concentration of 0.4% were added before use] (Miller et al., 1992) incubated overnight at 37°C with shaking at 200 rpm. On the next day, the overnight starter cultures were diluted 1:100 to inoculate 58 ml of the same medium in 50 ml falcon centrifuge tubes and incubated at 37°C with shaking at 200 rpm until the OD_600_ reached 0.3. The falcon centrifuge tubes were filled to the top with 58 ml medium in order to create an anaerobic atmosphere as far as possible. Please note that some residual oxygen availability cannot be excluded as polypropylene tubes are potentially allowing some minimal oxygen diffusion into the sample (Karian, 2003). A total of 8 samples, 4 palladium-treated and 4 controls, were prepared. Ammonium sulfate, potassium phosphate, ferrous sulfate heptahydrate, potassium hydroxide, magnesium sulfate heptahydrate, and D(+)-Glucose were purchased from Merck Life Science AS, Oslo, Norway.

### Determination of Palladium Toxicity

The lethality of Pd to *E. coli* was measured using the alamarBlue^TM^ Cell Viability Kit (Life Technologies AS, Oslo, Norway). The AlamarBlue^TM^ Cell Viability kit uses the reducing power of living cells to quantitatively measure the metabolic activity of bacteria. Resazurin, the active compound of the kit, is a cell permeable compound that is blue in color and non-fluorescent. Upon entering living cells, resazurin is reduced to resorufin, a compound that is red in color and highly fluorescent. Viable cells continuously convert resazurin to resorufin, increasing the overall fluorescence and color of the media surrounding cells.

Two hundred microliter of *E. coli* bacterial suspension of OD_600_ 0.3 in M63 minimal medium were mixed with different Pd concentrations (0.1, 1, and 10 mM) in a 96-well transparent plate. Immediately after that, the cell suspensions were mixed with 10 μl of the resazurin reagent (1 mg/ml stock concentration, which corresponds to 4 mM). Three controls with the same concentrations of Pd in 200 μl M63 medium were also mixed with 10 μl resazurin reagent and used for determination of fluorescence background. Then, the fluorescence was measured every 10 min continuously for 8 h at 37°C using a SYNERGY H1 microplate reader (BioTek, United States). The measurements were fluorescence endpoint with excitation at 530 nm, emission at 590 nm, and 100 gain. The intensity of fluorescence is proportional to the number of living cells and corresponds to the metabolic activity. Fluorescence intensity values (after the subtraction of fluorescence background) were used for the comparison of cell viability between different samples.

### Palladium Challenge

When the OD_600_ reached 0.3, 4 biological replicates were challenged with 100 μM Pd^2+^ in the form of sodium tetrachloropalladate (II) dissolved in 0.01 M nitric acid (similar amounts of Milli-Q water were added to the 4 control replicates). The pH of the samples was not affected (data not shown). The cultures were incubated at 37°C with shaking at 200 rpm for 10 min. The cells were harvested by centrifuging at 4200 × *g* for 10 min at 4°C. Then, the pellets were resuspended in 1 ml of 20 mM MOPS buffer (pH 7) and transferred to 2 ml Eppendorf tubes to remove excess heavy metal ions that might impact the subsequent RNA isolation and analysis. The tubes were centrifuged at 6000 × *g* for 2 min at 4°C. The supernatant was removed, and the tubes were immediately dipped into liquid nitrogen, stored at −80°C, and later shipped on dry ice. Sodium tetrachloropalladate (II) 98%, 5.6 M nitric acid, and MOPS were purchased from Merck Life Science AS, Oslo, Norway.

### RNA Sequencing

Total RNA isolation, RNA quality control, library preparation, sequencing, and data analysis were performed by Eurofins Scientific, Konstanz, Germany (INVIEW Transcriptome Bacteria package). Eurofins protocol contained the following steps: total RNA isolation by RNeasy kit (Qiagen, Hilden, Germany), rRNA depletion by NEBNext kit (New England Biolabs, Frankfurt, Germany), and RNA quality measurement by a fragment analyzer (Agilent Technologies, Ratingen, Germany). Then, the mRNA was fragmented and random hexamer primers were used for cDNA synthesis. Adapter ligation and adapter-specific PCR amplification were used to generate libraires of 150 bp reads. More than 10 million reads were generated per sample. The reads were paired-end sequenced by an Illumina sequencing platform.

### Data Analysis

The reads were mapped by Eurofins Scientific against the *E. coli* K-12 MG1655 strain reference genome (the closest *E. coli* reference genome to *E. coli* K-12 BW25113 genome) using the software BWA-MEM (version 0.7.12-r1039) (Li, 2013). Transcripts were identified and quantified. Then, pairwise comparison of expression levels and statistical analysis were carried out.

Raw read counts were created using featureCounts (version 1.5.1) (Liao et al., 2014). Only reads overlapping “CDS” features in the reference genome were counted. All reads mapping to features with the same meta-feature attribute were summed. Only reads with unique mapping positions and a mapping quality score of at least 10 were considered for read counting. Supplementary alignments were ignored for read counting. Paired-end reads that mapped with unexpected strandedness were ignored. Reads mapping to multiple features were assigned to the feature that has the largest number of overlapping bases.

A Trimmed Mean of M-values (TMM) normalization was performed using the edgeR package (version 3.16.5) (Robinson et al., 2009; Robinson and Oshlack, 2010). The basic assumption of this normalization technique is that most features (e.g., genes) should not be differentially expressed between samples. For each sample a normalization factor is calculated as the weighted mean of feature-wise log ratios between this sample and a reference sample.

### Bioinformatics Analysis

For the interpretation of the results based on gene functions, the differentially expressed genes with *p*-value < 0.05 were manually categorized by Clusters of Orthologous Groups (COGs) using NCBI COGs database^2^. Next, the differentially expressed genes were furtherly manually subcategorized based on their encoded functions into separate biological processes and pathways by Gene Ontology (GO) terms using UniProt^3^ and Quick GO databases^4^, and BioCyc pathway/genome database collection^5^.

### Genome-Scale Metabolic Modeling

We applied gene-expression flux balance analysis (gx-FBA) methodology (Navid and Almaas, 2012) to the genome-scale metabolic model *E. coli i*ML1515 (Monk et al., 2017) in Matlab 2020a (The Math Works Inc., 1991) using Cobra toolbox v3 (Heirendt et al., 2019) with Gurobi (Gurobi Optimization, 2014) as solver. The gx-FBA method integrates gene expression data with the *i*ML1515 model, allowing for response-modeling of environmental perturbations. In short, the cellular metabolic fluxes are scaled in relation to the mRNA expression data in the stress state of the cell. By optimizing for similarity between differentially expressed genes (and hence reaction fluxes) and the provided transcriptomics data, a flux distribution consistent with the constraints and gene expression values of the stressed state is calculated. The flux distribution of the reference state can then be compared with the flux distribution of the stress state. The predicted relative flux changes as a response to Pd stress are used as proxies for metabolic responses.

For modeling the cellular environment corresponding to the biocide environments, we allowed aerobic growth on the default *i*ML1515 medium, using glucose as the sole carbon source. For modeling the cellular environment corresponding to the Pd-stress environment, we set conditions to anaerobic growth and restricted carbon, phosphate and sulfate sources only to those available in the biomass (proteins and glycogen) in minimal amounts necessary to ensure survival, representing the cell using its reserves simply to satisfy ATP maintenance requirements. Secretion fluxes were not changed in the model. Reactions regulated by non-differentially expressed genes are not directly limited in their flux as they are not part of the gx-FBA objective function, but are nonetheless restricted by substrate availability (and ability for other reactions to consume their products), and therefore, indirectly restricted according to constraints applied to the reaction system. Given a set of constraints, which in our case is imposed by nutrient availability, gene expression, non-depletion/accumulation of metabolites and stoichiometry, an FBA optimal solution (including gx-FBA) will typically not be unique; rather, multiple different flux solutions might satisfy the objective function equally well. Therefore, flux values for a reaction are not necessarily fixed, but can be said to fall between a pair of upper and lower bounds, with some degree of uncertainty in the flux predictions given by a model.

In each of the gx-FBA simulations, we classify reactions into four categories. The first category, which we call “no flux,” consists of reactions which do not carry flux in the unstressed environment and are also unable to carry flux in stress conditions. The second category, “ambiguous,” consists of reactions for which the unstressed reaction flux falls between the upper and lower bounds returned by the gx-FBA optimization, meaning that up- or down-regulation of the flux is not a necessary consequence of the gene expression values. The last two categories are down-regulated and up-regulated reaction fluxes. Down-regulated fluxes are reaction fluxes for which the upper bound in the gx-FBA solution is smaller than the unstressed reaction flux, and which must therefore necessarily carry lower reaction flux in stress conditions. Conversely, up-regulated reaction fluxes are those for which the lower bound of the gx-FBA solution are larger than the unstressed reaction flux, which means that the expression values in stress conditions necessarily require an increase in the flux.

## Results

### Palladium Sublethal Concentration

**FIGURE 1:**
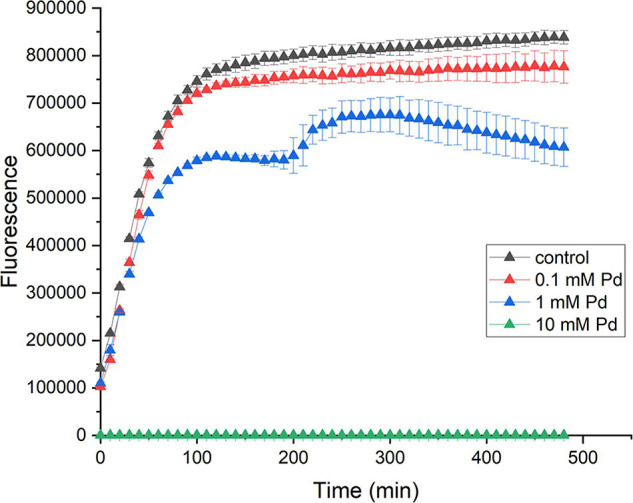
Effect of Pd on the viability on *E. coli*. *E. coli* cells were incubated with 10 mM (green triangles), 1 mM (blue triangles), and 0.1 mM (red triangles) sodium tetrachloropalladate (II) or with water as a control (black triangles). The viability of cells was measured for 8 h. The error bars represent the standard deviation of the mean for three replicates.

We have tested several Pd concentrations. Out of the three Pd concentrations used in the viability assay, the highest one (10 mM Pd) resulted in the complete inhibition of metabolism (the green graph in Figure 1). For 1 mM Pd, the cells seemed to be adversely impacted by this concentration, as the fluorescence signal was 22% less than the control in average. After 200 min of incubation at this concentration, the cells went through a phase of adaptation (the shoulder in the blue graph in Figure 1) for ca. 100 min. After that the cells start dying fast. For 0.1 mM Pd, the cells did not appear to be strongly affected, the reduction in fluorescence signal was 7% (the red graph in Figure 1). This concentration was used for Pd exposure in this study.

### A Transcriptomic View of Pd Stress

The RNA-Seq results showed that 709 genes, which account for 18.2% of the genes mapped in our experiment, were differentially expressed after 10 min of exposure to 100 μM Pd (*p*-value < 0.05). From those genes, 411 (58%) were up-regulated and 298 (42%) were down-regulated. The results are summarized in Table 1.

**TABLE 1:** Number of genes that were differentially expressed after 10 min of exposure to 100 μM Pd.

**Category**	**Number**	**Percentage**
Non-differentially expressed genes	3189	81.8%
Differentially expressed genes	709	18.2%
Up-regulated	411	58%
Down-regulated	298	42%
The cut-off *p*-value was 0.05		

*From 100% differentially expressed genes 58% were up-regulated and 42% were down-regulated.*

### Differentially Expressed Genes Grouped by Functional Categories

**FIGURE 2:**
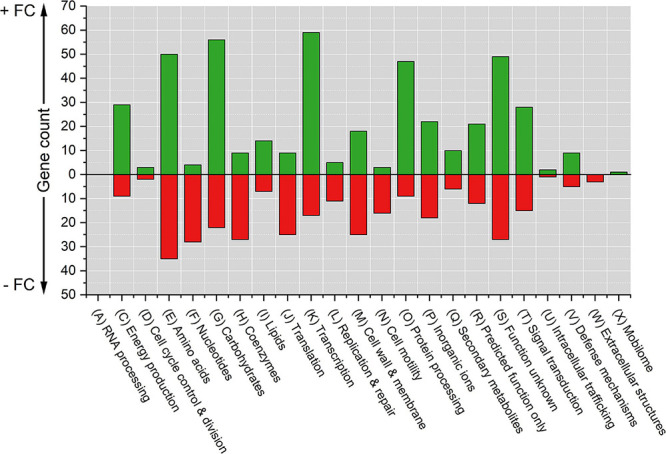
Counts of differentially expressed genes due to Pd stress grouped by COG functional categories. The cut-off *p*-value was 0.05. Positive counts represent observed up-regulated genes (green bars) and negative counts represent observed down-regulated genes (red bars). COG code, number of proteins encoded by *E. coli* genome and category description: (A, 3) RNA processing and modification; (C, 284) Energy production and conversion; (D, 43) Cell cycle control, cell division, and chromosome partitioning; (E, 356) Amino acid transport and metabolism; (F, 107) Nucleotide transport and metabolism; (G, 384) Carbohydrate transport and metabolism; (H, 180) Coenzyme transport and metabolism; (I, 122) Lipid transport and metabolism; (J,236) Translation, ribosomal structure and biogenesis; (K, 301) Transcription; (L, 140) Replication, recombination, and repair; (M, 254) Cell wall, membrane, and envelop biogenesis; (N,110) Cell motility; (O, 178) Post-translational modification, protein turnover, and chaperones; (P, 223) Inorganic ion transport and metabolism; (Q, 72) Secondary metabolites biosynthesis, transport, and catabolism; (R, 261) General function prediction only; (S, 255) Function unknown; (T, 193) Signal transduction mechanisms; (U, 50) Intracellular trafficking, secretion, and vesicular transport; (V, 93) Defense mechanisms; (W, 31) Extracellular structures; and (X, 60) Mobilome, prophages, and transposons.

The differentially expressed genes were grouped based on Clusters of Orthologous Groups (COGs) to identify expression differences based on gene functions (Figure 2 and Supplementary Data Sheet 1). Raw RNA-Seq data can be found in Supplementary Data Sheet 2, Tabs 1-28. In this paper, the COG category names are used together with their international one-letter code (Tatusov et al., 2000).

The top five COG categories with the highest number of differentially expressed genes were amino acid transport and metabolism (D) with 85 genes (50 up-regulated and 35 down-regulated); carbohydrate transport and metabolism (G) with 78 genes (56 up-regulated and 22 down-regulated); transcription (K) with 76 genes (59 up-regulated and 17 down-regulated); genes of unknown function (S) with 76 genes (49 up-regulated and 27 down-regulated); and post-translational modification, protein turnover, and chaperones (O) with 56 genes (47 up-regulated and 9 down-regulated). The five COG categories with the lowest number of differentially expressed genes were RNA processing and modification (A) with 0 genes affected; mobilome, prophages, and transposons (X) with 1 up-regulated gene; extracellular structures (W) with 3 down-regulated genes; intracellular trafficking, secretion, and vesicular transport (U) with 3 genes (2 up-regulated and 1 down-regulated); and cell cycle control, cell division, and chromosome partitioning (D) with 5 genes (3 up-regulated and 2 down-regulated).

The functional categorization of differentially expressed genes showed that there were categories that had a high up-regulation trend [energy production and conversion (C), transcription (K), and post-translational modification, protein turnover, and chaperones (O)] and other categories with a high down-regulation trend [nucleotide transport and metabolism (F), coenzyme transport and metabolism (H), translation, ribosomal structure and biogenesis (J), and cell motility (N)].

### Top Differentially Expressed Genes

The majority of differentially expressed genes (640 out 709 genes) had an absolute fold change (FC) value of 3.5-30 compared to the control (Supplementary Data Sheet 1). Tables 2, 3 show the top 20 highest up- and down-regulated genes, respectively.

**TABLE 2:** Top 20 most up-regulated genes after 10 min of exposure to 100 μM Pd.

**Gene ID**	**Gene name**	**Product description**	**FC**	**Error**
b1743	*spy*	Periplasmic ATP-independent protein refolding chaperone	6447	± 2830
b1970	*hiuH*	5-hydroxyisourate hydrolase, zinc metal-binding	1465	± 986
b3686	*ibpB*	Chaperone, heat shock protein of HSP20 family	565	± 94
b4002	*zraP*	Important component of the zinc-balancing mechanism	357	± 45
b4484	*cpxP*	Part of the cpx two-component envelope stress response system	330	± 92
b4062	*soxS*	Transcriptional activator of the superoxide response regulon	240	± 28
b2074	*mdtA*	Part of *mdt*ABC-*tol*C efflux pump, multidrug resistance	188	± 47
b4140	*fxsA*	Suppresses F exclusion of phage T7	163	± 35
b3263	*yhdU*	Function unknown	160	± 148
b1742	*ves*	Cold and stress-inducible protein	151	± 88
b2162	*rihB*	Pyrimidine-specific ribonucleoside hydrolase	133	± 17
b3501	*arsR*	Arsenate resistance operon repressor	132	± 20
b1972	*yedZ*	Protein-methionine-sulfoxide reductase heme-binding subunit MsrQ	112	± 15
b0484	*copA*	Copper-, silver-translocating ATPase efflux pump; involved in copper resistance	101	± 10
b3828	*metR*	Positive activator of the metA, metE and metH genes	100	± 4
b1971	*yedY*	Protein-methionine-sulfoxide reductase catalytic subunit MsrP	96	± 8
b3708	*tnaA*	Tryptophanase, synthesis of pyruvate from L-tryptophan pathway	94	± 3
b4670	*yjeV*	Function unknown	81	± 63
b3502	*arsB*	Arsenite pump; resistance to arsenate, arsenite, and antimonite	76	± 8
b3687	*ibpA*	Chaperone, heat-inducible protein of HSP20 family	70	± 7

**TABLE 3:** Top 20 most down-regulated genes after 10 min of exposure to 100 μM Pd.

**Gene ID**	**Gene name**	**Product description**	**FC**	**Error**
b2725	*hycA*	Regulatory protein for the formate hydrogenlyase system	−520	± 151
b1038	*csgF*	Curli production assembly/transport component	−338	± 60
b3215	*yhcA*	Required for the biogenesis of putative fimbria	−197	± 32
b0336	*codB*	Required for cytosine transport into the cell	−130	± 23
b2145	*yeiS*	Function unknown	−130	± 103
b2269	*elaD*	Protease, capable of cleaving an AMC-ubiquitin model substrate	−119	± 13
b4665	*ibsC*	Toxic component of a type I toxin-antitoxin (TA) system	−117	± 28
b2345	*yfdF*	Function unknown	−117	± 19
b4128	*ghoS*	Antitoxin component of a type V toxin-antitoxin (TA) system	−110	± 12
b1520	*yneE*	Predicted inner membrane protein, bestrophin family; possible chloride channel	−109	± 27
b0945	*pyrD*	Dihydroorotate dehydrogenase, UMP biosynthesis	−106	± 21
b3622	*waaL*	LPS core biosynthesis; O-antigen ligase	−85	± 13
b0991	*ymcE*	Cold shock protein, function unknown	−80	± 64
b2352	*gtrS*	Serotype-specific glucosyl transferase, CPS-53/KpLE1 prophage	−70	± 16
b2497	*uraA*	Uracil permease, uracil transport into the cell	−59	± 4
b2669	*stpA*	RNA chaperone and DNA-binding protein	−50	± 14
b0564	*appY*	Induces the synthesis of acid phosphatase (AppA) and several other polypeptides	−49	± 23
b4345	*mcrC*	McrBC restriction endonuclease	−44	± 5
b3508	*yhiD*	Putative magnesium transporter	−41	± 16
b1018	*efeO*	Involved in Fe^2+^ uptake	−41	± 7

### Detailed Results of Differentially Expressed Genes Based on Clusters of Orthologous Groups Categorization and Gene Ontology Terms

#### Genes Related to Information Macromolecules

##### DNA replication, recombination, and repair (L)

Of the genes responsible for initiation, maintenance, and termination of chromosome replication, only one gene was down-regulated (*recQ*) and one was up-regulated (*smbC*). Of the genes encoding polymerase subunits, no genes were differentially expressed. Finally, of the genes responsible for DNA repair and recombination, 8 genes were down-regulated and 2 were up-regulated. Detailed information can be found in Supplementary Data Sheet 3, Tab 1.

##### Transcription (K)

The genes for RNA polymerase subunits were not affected by Pd exposure. The same applies to the termination factors and degradosome complex genes. Of the genes responsible for RNA processing enzymes and RNases, 2 were down-regulated (*ghoS* and *rnb*). Three out of the 7 sigma factors were differentially expressed, FecI (ferric citrate sigma factor) was down-regulated, RpoS (stationary phase and stress response sigma factor) and RpoH (heat shock sigma factor) were up-regulated. Detailed information can be found in Supplementary Data Sheet 3, Tab 2. Many transcriptional regulators were differentially expressed due to Pd exposure, 50 were up-regulated and 10 were down-regulated (Supplementary Data Sheet 3, Tab 3). Of all COG categories, transcription had the highest number of up-regulated genes (59 genes).

##### Translation, ribosomal structure and biogenesis (J)

Six out of the genes responsible for ribosome-assembly proteins (r-proteins) were down-regulated, 5 of them were from the *rpsJ* operon. Of the other r-proteins, 7 gene were down-regulated (several methyltransferases) and 4 were up-regulated (stress-related proteins responsible for ribosome stabilization). Moreover, 3 genes responsible for ribosome recycling and stalled-ribosome rescue were up-regulated. The translation initiation and termination factors were unchanged, while 2 of the elongation factors were down-regulated. For the genes of tRNA synthetases/ligases, only one was down-regulated (proline-tRNA ligase). While for tRNA processing genes, 8 were down-regulated and 2 were up-regulated. Detailed information can be found in Supplementary Data Sheet 3, Tab 4. This shows that the ribosome assembly process and translation overall is repressed due to Pd stress, while at the same time genes for recycling and rescuing present ribosomes are up-regulated.

##### Post-translational modification, protein turnover, and chaperones (O)

Intoxication with several heavy metals can cause protein cross-linking (Rouch et al., 1995), which disrupts their 3D structures and allosteric movements. The proteases and chaperones of the heat shock response repair or degrade misfolded proteins (Georgopoulos and Welch, 1993). Pd stress induced these repair mechanisms, as 28 different genes for proteases, heat shock proteins, and chaperones were highly up-regulated. Five of them were in the top 20 most up-regulated genes, namely *spy* (6447-fold), *ibpB* (565-fold), *cpxP* (330-fold), *ves* (151-fold), and *ibpA* (70-fold). Of the RNases and RNA processing enzyme genes, only one was down-regulated (*rnb* RNase II, responsible for mRNA degradation). Detailed information can be found in Supplementary Data Sheet 3, Tab 5.

#### Genes Related to Central Metabolism

##### Carbohydrate transport and metabolism (G)

This COG category had the 2nd highest number of up-regulated genes (56 genes). Most of the up-regulated genes are related to carbohydrate transport. For the transport of disaccharides, *malEKX* and the regulator gene *mlc* involved in maltose transporter complex; *mglA* and *mglB* involved in galactose transporter complex; and *treB* and *treC* involved in trehalose transport and hydrolysis systems, respectively, were up-regulated. For the transport of hexoses, *frwA* fructose transporter gene; *gntP* gluconate transporter gene; *ytfQRT* galactofuranose transporter genes; *srlABE* sorbitol transporter genes; *manXY* mannose transporter genes; *garP* galactarate transporter gene; and L-fucose-proton symporter gene *fucP* were all up-regulated. The expression of some hexoses metabolic enzymes was also increased. All the genes (*fucAIKOR*) from L-fucose degradation pathway to make L-lactate and pyruvate; *dgoADK* genes involved in making pyruvate from D-galactonate; and *garD* involved in galactarate degradation pathway to make pyruvate were up-regulated.

For the transport of pentoses, *xylF* xylose transporter gene; *rbsA* and *rbsB* involved in the ribose uptake system; and *araF* L-arabinose transporter gene were up-regulated. The sugar efflux transporter gene *ydeA* responsible for L-arabinose level maintenance was down-regulated. In addition to transport, several genes from pentose and glucuronate interconversion pathways were up-regulated (*uxaABC* and *uxuAB*). For the transport of other kinds of sugars, *glpT* and *glpF* glycerol transporter genes and *lsrABCDK* from autoinducer 2 carbohydrate import system were up-regulated. The function of *lsr* system is not clear, but it has been suggested to have functions in quorum sensing (Xavier and Bassler, 2005).

The other genes that were down-regulated were from unconnected parts of pathways or were poorly defined. Detailed information can be found in Supplementary Data Sheet 4, Tab 1. The systematic up-regulation of carbohydrate transport genes suggests that the cells were in high demand for sugars as they were trying to make energy through different pathways simultaneously to cope with Pd stress.

##### Energy production and conversion (C)

**FIGURE 3:**
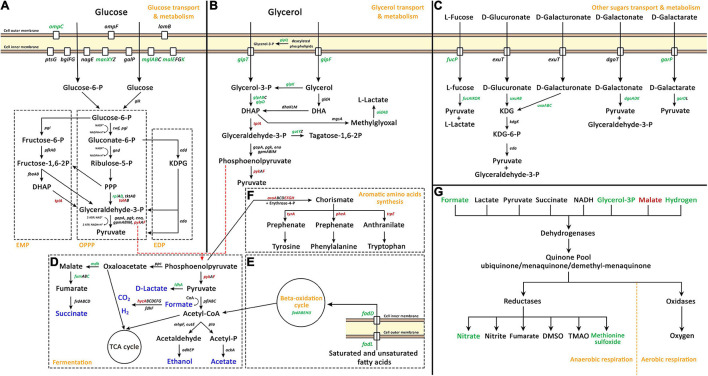
Palladium (Pd) stress effects on genes related to carbohydrate transport, carbohydrate metabolism and energy production pathways, namely, glucose transport and metabolism **(A)**, glycerol transport and metabolism **(B)**, other sugars transport and metabolism **(C)**, fermentation **(D)**, beta-oxidation cycle **(E)**, aromatic amino acids synthesis **(F)**, and anaerobic respiration **(G)**. In panels **(A–F)**, green gene names represent up-regulated genes, red names represent down-regulated genes, and black names represent unaffected genes. In panel **(D)**, blue color represents fermentation end products. In panel **(G)**, green and red colors represent up-regulated and down-regulated parts of anaerobic respiration pathways, respectively. In panel **(A)**, the three native glycolytic pathways in *E. coli* are demonstrated: Embden-Meyerhof pathway (EMP), oxidative pentose-phosphate pathway (OPPP), and Entner-Doudoroff pathway (EDP). The full names of abbreviated compounds are DHAP, dihydroxyacetone phosphate; KDPG, 2-Keto-3-deoxy-6-phosphogluconate; DHA, dihydroxyacetone; KDG, 2-dehydro-3-deoxy-D-gluconate; TCA, tricarboxylic acid cycle; DMSO, dimethyl sulfoxide; and TMAO, Trimethylamine N-oxide.

Several metabolic processes related to energy production were highly modulated upon Pd exposure (shown in detail in Figure 3). For glucose transport and metabolism (Figure 3A), glucose transport was up-regulated through the up-regulation of one the outer membrane porins, OmpC, and three of the seven glucose transporter systems (Alva et al., 2020). *E. coli* has three native glycolytic pathways: the Embden-Meyerhof pathway (EMP), the oxidative pentose-phosphate pathway (OPPP), and the Entner-Doudoroff pathway (EDP) (Hollinshead et al., 2016). None of these pathways were highly modulated, but one enzyme from the EMP, TpiA, was down-regulated. For OPPP, 2 enzymes were down-regulated, TalA and PykF, and one was up-regulated, RpiB. Interestingly, the expression of the genes responsible for the transport and metabolism of glycerol (Figure 3B) and other sugars (Figure 3C) was highly increased despite the fact that these sugars were not present in the culture medium. Glycerol transport and metabolism genes had a high up-regulated trend; its two transport systems, GlpQ enzyme (glycerophosphoryl diester phosphodiesterase which hydrolyzes deacylated phospholipids to glycerol-3-phosphate), and several of its metabolic enzymes were up-regulated. For the transport and metabolism of other sugars, five sugar degradation pathways to make pyruvate were up-regulated (from L-fucose, D-glucuronate, D-galacturonate, D-galactonate, and D-galactarate) together with the transporters of L-fucose and D-galactarate.

Fermentation processes (Figure 3D) were also modulated. The production of D-lactate, malate, and fumarate was increase thought the up-regulation of *ldhA*, *mdh*, and *fumC*, respectively. The production of H_2_ was highly modulated thought the massive down-regulation of the transcriptional repressor of hydrogenase 3 operon, HycA, which was the most down-regulated gene due to Pd exposure, decreasing 520-fold. Moreover, two other pathways that are related to fermentation were highly modulated. The beta-oxidation cycle (Figure 3E) that produces acetyl-CoA was highly up-regulated through the up-regulation of the outer membrane protein FadL and the inner membrane protein FadD responsible for the transport of saturated and unsaturated fatty acids, together with the beta-oxidation cycle genes, *fadABEHIJ*.

Phosphoenolpyruvate enters two separate pathways; it is either converted to oxaloacetate or to pyruvate. In addition, phosphoenolpyruvate is also the precursor for aromatic amino acids synthesis (Figure 3F) together with erythrose-4-P. This pathway was massively down-regulated on two levels, firstly, through the down-regulation of *aroAEFGH* genes which encode enzymes that convert phosphoenolpyruvate and erythrose-4-P to chorismate, and secondly, through the down-regulation of aromatic amino acids individual pathways, through *tyrA*, *pheA*, and *trpE* genes.

Besides the modulation of glycolysis and fermentation processes, the anaerobic respiration process was also modulated. In this study, HNO_3_ was used a solvent for sodium tetrachloropalladate (II). The addition of HNO_3_ (0.291 μL from 0.01 M stock concentration to 58 mL medium) created a 50 μM HNO_3_ concentration in the medium. *E. coli* can use the nitrate of HNO_3_ as terminal electron acceptor for anaerobic respiration. The nitrate reductase operons of *E. coli* are optimally expressed at higher nitrate concentrations. Genes such as *napF*, *nrfA*, and *nirB* are optimally expressed at 1 mM nitrate while *narG* is optimally expressed at 10 mM nitrate concentration (Wang et al., 1999; Wang and Gunsalus, 2000). However, the minimal HNO_3_ concentration added might be the cause for this modulation, at least partially (Figure 3G).

For respiration in *E. coli*, dehydrogenases transfer electrons from various substrates to the quinone pool. Then, terminal reductases and oxidases transfer these electrons to different electron acceptors. For aerobic respiration, the terminal electron acceptor is O_2_, while for anaerobic respiration there are different electron acceptors. On the dehydrogenases level, the expression of formate and glycerol-3-P dehydrogenases and the expression of hydrogenase 2 was increased, while the expression of malate dehydrogenase was decreased. On the reductases level, the expression of nitrate and methionine sulfoxide reductases was up-regulated.

In addition to all this, the expression of the glucose dehydrogenase gene *gcd* and 4 of the 6 genes of the *rsx* operon (ion-translocating oxidoreductase complex) were down-regulated. This complex is a membrane-bound complex that couples electron transfer with translocation of protons across the membrane. Detailed information about energy production and conversion modulated genes can be found in Supplementary Data Sheet 4, Tab 2.

##### Amino acid transport and metabolism (E)

**FIGURE 4:**
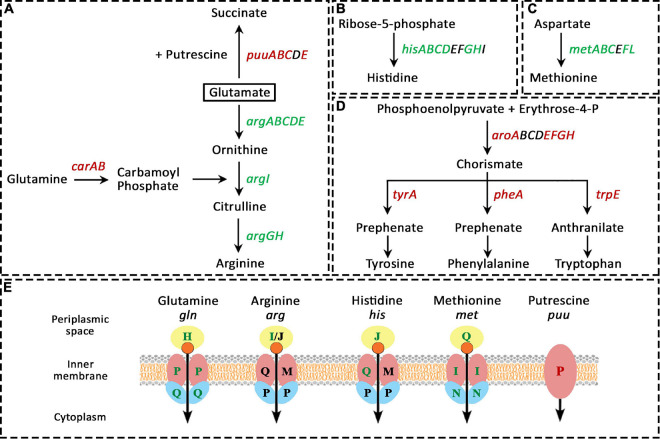
Amino acid-related pathways that were heavily modulated due to Pd stress. In panels **(A–D)**, green gene names represent up-regulated genes, red names represent down-regulated genes, and black names represent unaffected genes. In figure **(E)**, green, red, and black letters represent up-regulated, down-regulated, and unaffected genes, respectively. **(A)**, arginine biosynthesis from glutamate (*argABCDEGHI*) was highly up-regulated, but the biosynthesis of arginine from glutamine was down-regulated (*carA* and *carB* genes). The conversion of glutamate and putrescine to succinate by the products *puuABCDE* genes was also heavily down-regulated. The biosynthesis of histidine **(B)** and methionine **(C)** was also highly up-regulated. **(D)**, the biosynthesis of aromatic amino acids was drastically down-regulated on two levels, firstly, thought the down-regulation of the production of their common precursor (chorismate) from erythrose-4-phosphate by the products of *aroABCDEFGH* genes, and secondly, through the down-regulation of their individual pathways. **(E)**, the pathways involved in the transport of glutamine, arginine, histidine, and methionine were up-regulated. Lastly, the putrescine importer gene *puuP* was down-regulated.

This category of COGs was modulated the most under Pd stress with 85 genes being differentially expressed, 50 up-regulated and 35 down-regulated. We found particular amino acid pathways that were heavily modulated (more than 5 genes of the pathway), others that were slightly modulated with 2-3 genes being affected, and the rest had only one or no genes being modulated. The amino acid synthesis pathways that were heavily modulated are shown in Figure 4. The modulation is divided into two parts: biosynthesis pathways and uptake pathways. On the biosynthesis level, arginine biosynthesis was highly up-regulated, with all the 8 genes involved in its biosynthesis from glutamate (*argABCDEGHI*) were up-regulated. Arginine could alternatively be synthesized from glutamine by converting glutamine to carbamoyl phosphate by the products of the *carA* and *carB* genes; but we found this pathway was down-regulated. Glutamate together with putrescine can be also converted to succinate by the products of *puuABCDE* genes, this pathway was dramatically down-regulated. It appears that bacteria are saving all the glutamate for arginine biosynthesis, and particularly through this pathway and not from glutamine (Figure 4A).

The biosynthesis of histidine (Figure 4B) and methionine (Figure 4C) was also highly up-regulated, where the genes *hisABCDGH* and *metABCFL* were up-regulated, respectively. Moreover, the expression of the positive regulator of the methionine operon, MetR, increased 100-fold. At the same time, the biosynthesis of aromatic amino acids was severely down-regulated. For their biosynthesis, phosphoenolpyruvate and erythrose-4-P have to be converted to chorismate by the products of *aroABCDEFGH* genes, then, chorismate can be converted to the three aromatic amino acids through three different pathways. For tyrosine and phenylalanine, the first step in their biosynthesis is that chorismate is converted to prephenate by the products of *tyrA* and *pheA* genes, respectively, while for tryptophan, chorismate is converted to anthranilate by the product of *trpE* gene. The majority of all these genes were down-regulated (Figure 4D).

On the uptake level (Figure 4E), a comparable behavior was found. The pathways involved in the transport of glutamine, arginine, histidine, and methionine were up-regulated as well. For glutamine and methionine, all the three genes involved in their transporter complex were up-regulated (the periplasmic binding protein (GlnH and MetQ, respectively), the membrane permease (GlnP and MetI, respectively), and the ATP-binding import protein [GlnQ and MetN, respectively)]. For histidine, the genes for the periplasmic binding protein HisJ and the permease HisQ were up-regulated. For arginine, only the periplasmic binding protein ArgI was up-regulated. Lastly, the putrescine importer PuuP was down-regulated.

For the amino acid pathways that were slightly modulated, 2 genes involved in the serine biosynthesis pathway were down-regulated, *serA* and *serC*. Two genes involved in the isoleucine biosynthesis pathway were down-regulated, *ilvC* and *ilvD*. The three genes involved in the transporter complex of glycine betaine, *yehWXY*, were down-regulated. The genes encoding the S-methylmethionine permease, *mmuP*, and homocysteine S-methyltransferase, *mmuM*, were up-regulated. Lastly, two genes involved in the catabolism of tryptophan, *tnaA* and *tnaB*, were up-regulated. Detailed information can be found in Supplementary Data Sheet 5, Tab 1.

##### Inorganic ion transport and metallochaperones (P)

**FIGURE 5:**
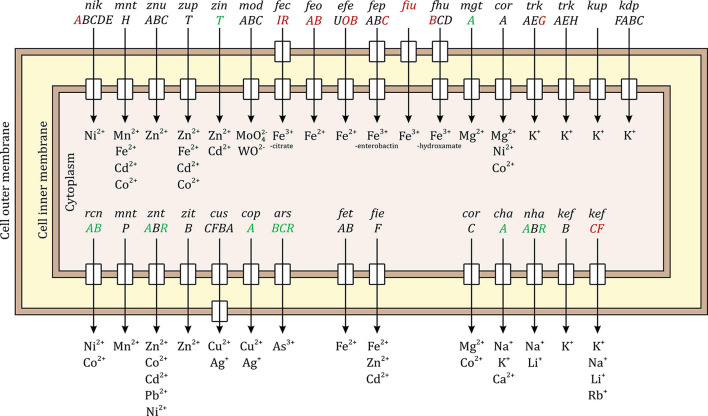
Palladium (Pd) stress effects on inorganic metal ion import and export systems in *E. coli*. The upper half of the figure shows the systems responsible for ion import and the lower half shows the systems responsible for ion export. Some systems are specific to one metal ion while other can transport a variety of ions. The small rectangles in the inner membrane indicate that the system has an inner member permease protein and the rectangles in the outer membrane indicate that the system includes an outer membrane porin. The up-regulated genes due to Pd stress are shown in green, the down-regulated genes in red, and the unaffected genes in black. Ten of the importing systems were modulated, 8 of them were down-regulated. All the iron importing systems were down-regulated. Seven of the exporting systems were modulated, 6 of them were up-regulated.

*Escherichia coli* has several systems for importing, balancing, and exporting bulk and trace metal ions. Some of these systems are very specific to one metal ion while others can transport a variety of ions. Figure 5 shows the well-studied systems in *E. coli*, the upper half of the figure shows the systems responsible for ion import while the lower half shows the systems responsible for ion export. Ten of the 17 systems responsible for importing inorganic ions were differentially expressed, 8 down-regulated and 2 up-regulated. The periplasmic binding protein gene *nikA* of the nickel transporter complex operon *nikABCDE* was down-regulated. The uptake protein gene *trkG* of the potassium transporter complex operon *trkAEG* was down-regulated. Interestingly, all the 6 systems responsible for iron import were down-regulated, namely, the ferric citrate sigma factor gene *fecI* and the regulator gene *fecR* responsible for the *ficABCDE* operon of the Fe^3+^-citrate import system; the periplasmic binding protein gene *feoA* and the GTP-binding channel protein gene *feoB* of *feoABC* operon of the Fe^2+^ import system; the periplasmic binding protein gene *efeO* and the deferrochelatase gene *efeB* of the *efeUOB* operon of the Fe^2+^ import system; the ATP-binding protein gene *febC* of *fepABCDG* operon of the Fe^3+^-enterobactin import system; the siderophore receptor gene *fiu* responsible for iron transport across the outer membrane complexed with catecholate siderophores such as dihydroxybenzoylserine and dihydroxybenzoate; and the permease protein gene *fhuB* of *fhuBCD* operon of the Fe^3+^-hydroxamate import system. The up-regulated genes were the magnesium-transporting ATPase gene *mgtA* and periplasmic zinc chaperon gene *zinT*.

Seven of the 14 systems responsible for exporting inorganic ions were differentially expressed, 6 up-regulated and 1 down-regulated. The up-regulated genes were *rcnA* and *rcnB* of the nickel/cobalt efflux system; the transporting ATPase gene *zntA* and the regulator gene *zntR* of the zinc/cadmium/lead system; the copper-exporting ATPase gene *copA*; the channel protein gene *arsB*, arsenate reductase gene *arsC*, and the regulator gene *arsR* of the arsenical resistance system; the sodium-potassium/proton antiporter gene *chaA*; and the sodium/proton antiporter gene *nhaA* together with its regulator gene *nhaR*. The down-regulated genes were the glutathione-regulated potassium-efflux system gene *kefC* and its regulator gene *kefF*.

In addition to the transporter complexes, several other metal-related pathways were modulated as well. The genes involved in the biosynthesis of the enterobactin siderophore, a low-molecular mass compound that is secreted extracellularly by *E. coli* to chelate Fe^3+^ from the environment and then it is brought back to the cell through the FepABCDG system (Gehring et al., 1997), were down-regulated (*entC* and *entE*). The iron storage system was up-regulated through *ftnB* gene. All the genes of the *iscARSU* operon involved in Fe-S cluster assembly were up-regulated. The gene for copper oxidase, *cueO*, was highly up-regulated, increasing 44-fold.

Three other non-metal transporters were also modulated. The phosphate transporter complex permease gene *pstA* was down-regulated, the nitrate/nitrite transporter genes *narK* and *narU* were down-regulated, and the periplasmic binding protein gene *tauA* and the ATP-binding protein gene *tauB* of taurine import system were up-regulated. Detailed information can be found in Supplementary Data Sheet 5, Tab 2.

##### Coenzyme transport and metabolism (H)

This COG category had a high down-regulation trend, mainly on the vitamin biosynthesis level. Nine of the 11 genes involved in vitamin B1 (thiamine) biosynthesis were down-regulated (*thiCDEFGHIMS*). Two genes involved in vitamin B2 (riboflavin) biosynthesis pathway were down-regulated (*ribA* and *yigB*). For vitamin B3 (nicotinamide riboside), its transporter gene *pnuC*, and one gene involved in its biosynthesis (*pncB*) were down-regulated. Of the vitamin B6 (pyridoxal phosphate) biosynthesis pathway, 2 genes were down-regulated (*pdxK* and *pdxY*). Of the vitamin B9 (folate) biosynthesis pathway, 2 gene were down-regulated (*folE* and *folK*). For vitamin B12 (cobalamin), one gene of its transporter complex to the cell was down-regulated (the ATP-binding protein gene *btuD*), but one gene of its biosynthesis pathway was up-regulated (*btuR*).

In addition to vitamins, the synthesis of quinones was modulated. Two genes involved in ubiquinone biosynthesis pathway were up-regulated (*ubiA* and *ubiC*), but 2 genes involved in menaquinone/demethyl-menaquinone biosynthesis pathway were down-regulated (*menF* and *menH*). Moreover, the biosynthesis of iminosuccinic acid through the oxidation of aspartate was down-regulated through *nadB*. Phenylacetate degradation pathway was up-regulated through *paaJ* and *paaK*. Detailed information can be found in Supplementary Data Sheet 5, Tab 3.

##### Nucleotide transport and metabolism (F)

This COG category had a high down-regulation trend too, 28 genes were down-regulated (3 of them were in top 20 most down-regulated genes) compared to only 4 up-regulated genes. For purine biosynthesis pathway from phosphoribosyl pyrophosphate (PRPP), 9 genes were down-regulated, *purBCEFHKMNU*. For pyrimidine biosynthesis pathway from bicarbonate, 4 genes were down-regulated, *pyrBCDI*. For uridine monophosphate (UMP) biosynthesis pathway, 2 genes were down-regulated, *carA* and *carB*. Finally, for guanosine monophosphate (GMP) biosynthesis pathway, 2 genes were down-regulated, *guaA* and *guaB*. Of purine and pyrimidine hydrolysis pathways, 3 genes were drastically up-regulated, *rihB*, *rihC* and *hiuH*, the first being the 11th and last being the 2nd highest up-regulated genes with 133- and 1465-fold, respectively.

The genes involved for the biosynthesis of nucleotides through the nucleotide salvage pathway were also down-regulated: for adenosine monophosphate (AMP), *apt* was down-regulated; for cytidine monophosphate (CMP), *codA* was down-regulated; for GMP and xanthosine monophosphate (XMP), *gpt* was down-regulated; for inosine monophosphate (IMP), *gsk* was down-regulated; and for UMP, *upp* was down-regulated.

For the transport of nucleotides, all the permeases involved in the uptake of nucleobases were down-regulated. Adenine permease gene *adeP*, cytosine permease gene *codB*, guanine/hypoxanthine permease gene *ghxP*, uracil permease gene *uraA*, xanthine permease gene *xanP*, and the nucleoside permease gene *nupC*. Detailed information can be found in Supplementary Data Sheet 5, Tab 4.

##### Lipid transport and metabolism (I)

This category had a low number of modulated genes, 14 up-regulated and 7 down-regulated. The key pathway that was affected was the fatty acid beta-oxidation cycle, where 6 of its genes, *fadABEHIJ*, were up-regulated. The fatty acid transport protein genes *fadL* and *fadD* were also up-regulated. Two genes of isopentenyl diphosphate biosynthesis via DXP pathway, *ispD* and *ispF*, were down-regulated. Detailed information can be found in Supplementary Data Sheet 5, Tab 5.

#### Cell Cycle Control, Cell Division, and Chromosome Partitioning (D)

For this category, only a few genes were differentially expressed. The cell death peptidase gene *lit* and the cell division protein gene *yedR* were down-regulated. The toxic component gene *ibsC* of type I toxin-antitoxin (TA) system (Fozo et al., 2008) and the antitoxin component gene *ghoS* of type V TA system (Cheng et al., 2014) were heavily down-regulated, being the 7th and 9th most down-regulated, decreasing 117- and 110-fold, respectively. The toxic component gene *mokC* of the type I TA system (Pedersen and Gerdes, 1999) was up-regulated.

#### Cell Wall Functions

##### Cell wall biogenesis, defense mechanisms, and extracellular structures (M, V, and W)

For cell wall biogenesis, several genes involved in LPS biosynthesis were down-regulated, *arnC*, *eptC*, *IptD*, *lpxH*, *waaC*, *waaL* and *wza*. Several genes involved in enterobacterial common antigen biosynthesis pathway were also down-regulated, *rffG*, *rffH*, *wecB* and *wecC*. Moreover, *osmB* gene of the osmotically inducible lipoprotein B, which provides resistance to osmotic stress, was up-regulated.

More importantly, *E. coli* encodes multiple antibiotic efflux systems that increase drug efflux and limit passive uptake by decreasing porin expression (Alekshun and Levy, 1999). These genes are activated in response to antibiotics and general stress. We found that Pd stress activates them too. From the multiple antibiotic resistance MarRAB system, 3 out of 4 genes were highly up-regulated, *marABR*. Several TolC-dependent systems were modulated too. From the multidrug resistance MdtABC system, 5 genes were vastly up-regulated, *mdtABCDG*, *mdtA* was the 7th highest up-regulated gene, increasing 188-fold. While for the anaerobic multidrug efflux transporter genes *mdtEF*, *mdtE* was down-regulated. From the AcrAB system, 2 genes were up-regulated, *arcD* and *arcR*. From the EmrAB energy-dependent system, *emr*B was down-regulated. This might suggest that *E. coli* is trying to use the passive efflux systems and not the active ones, to save energy for other crucial systems. Finally, from the outer membrane porins (Omp) system, *ompC* was up-regulated. OmpC imports extracellular glucose to the periplasmic space (Alva et al., 2020), the high need for energy is possibly the reason for this up-regulation. Detailed information can be found in Supplementary Data Sheet 6, Tab 1.

##### Motility and biofilm (N)

This category had a heavy down-regulation trend with a total of 16 genes being down-regulated compared to only 3 up-regulated genes. Of the *flg* operon responsible for flagellar synthesis, 8 genes, *flgBCDEFGHI*, were down-regulated. The same effect was found on type 1 fimbriae, which are filamentous pili that are attached to the cell surface and mediate biofilm formation and adhesion onto host cells. *FimBCDFG*, encoding proteins responsible for the assembly of FimA fimbrial subunit, its export across the outer membrane, and its regulation, were all down-regulated. Moreover, *ecp*B was also down-regulated, which is a member of *ecpRABCDE* operon that encodes the *E. coli* common pilus (ECP) and plays a role in early-stage biofilm development and host cell recognition. In addition, genes involved in curli organelles biogenesis were heavily down-regulated, the expression of *csgE* and *csgF* was 16- and 338-fold lower, respectively. At the same time, the 3 genes that were up-regulated were *bssS* (a biofilm repressor gene), *tomB* (a biofilm formation regulator gene), and *glgS* (a negative regulator gene of motility, adhesion, and synthesis of biofilm exopolysaccharides). In summary, this all shows that the bacteria were intentionally avoiding the structurally and energetically intensive motility and biofilm formation systems. Detailed information can be found in Supplementary Data Sheet 6, Tab 2.

#### Stress Responses

##### Oxidative stress response and repair

**FIGURE 6:**
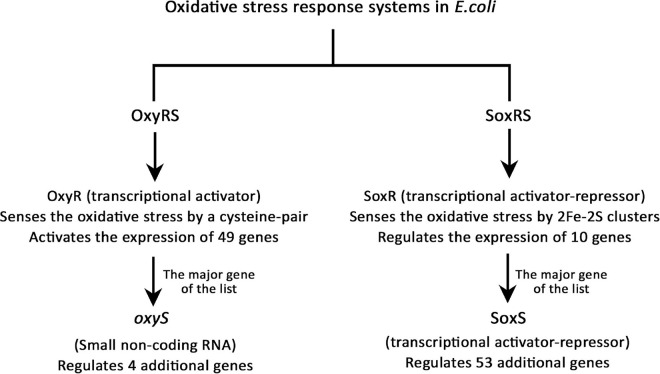
The oxidative stress response systems in *E. coli*.

There are two oxidative stress response systems in *E. coli*, the OxyRS and SoxRS regulons (Seo et al., 2015), explained in Figure 6. OxyR is a member of the LysR transcriptional regulators family, which uses a cysteine-pair to sense the oxidative damage and regulates 49 genes when oxidized (Choi et al., 2001). Nine genes out of those 49 were up-regulated due to Pd stress and no genes were down-regulated. *oxyS* is a small non-coding RNA (ncRNA) regulated by OxyR, which represses *rpoS* (stationary phase and stress response sigma factor gene), *fhlABC* flagellar proteins genes, and other genes to prevent redundant induction of stress response genes (Altuvia et al., 1998). Our RNA purification and library preparation methods were not optimized for the enrichment of nc-RNAs; therefore, we were not able to detect any of them. Despite being unable to detect the expression of *oxyS*, several genes regulated by it were modulated. The expression of *flhA* and *flhC* was down-regulated but the expression of *rpoS* was up-regulated. The up-regulation of *rpoS* might be a due to the low number of OxyR stress response activated genes (9 out of 49), which resulted in no redundant induction of stress response genes.

The SoxRS regulon is the other oxidative stress response system in *E. coli*. SoxR is a member of the MerR repressor-activator family, which uses the oxidation state of 2Fe-2S clusters to sense the oxidative damage and regulates 10 stress-related genes (Watanabe et al., 2008). The most important gene of SoxR regulated genes is *soxS*, which is responsible for the regulation of additional 53 genes (Seo et al., 2015). Out of the 10 genes regulated by SoxR, 4 genes were up-regulated and 3 were down-regulated. *soxS* was modulated the highest in this list, its expression was up-regulated 240-fold. Then, of the 53 genes regulated by SoxS, 15 genes were up-regulated and 4 were down-regulated. This altogether shows that Pd exposure caused a serious oxidative stress to *E. coli*. Detailed information can be found in Supplementary Data Sheet 7.

### Genome-Scale Metabolic Modeling

**FIGURE 7:**
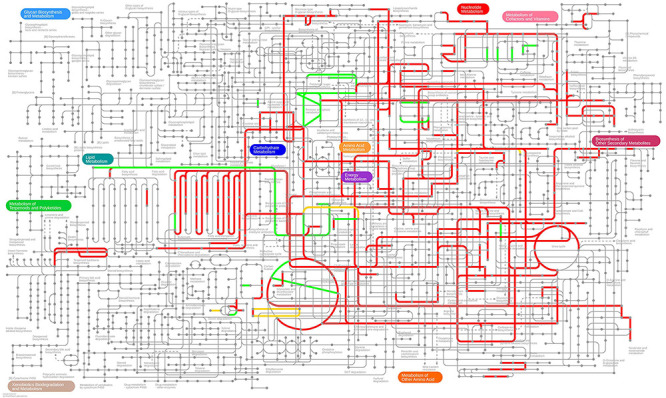
Predicted up- and down-regulated median fluxes across ten biocides (Pereira et al., 2020) using the *i*ML1515 model, presented in the KEGG pathway map of *E. coli*. Up-regulated fluxes are green, down regulated fluxes red, and fluxes without a regulation requirement are colored yellow. No-flux reactions and reactions without KEGG IDs are omitted.

**FIGURE 8:**
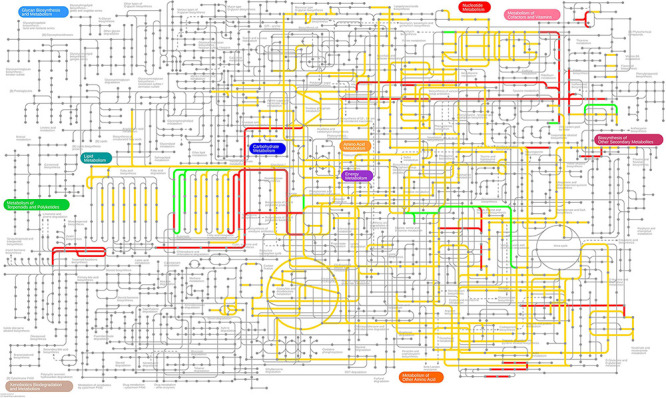
Predicted up- and down-regulated fluxes in response to Pd stress using the *i*ML1515 genome-scale metabolic model, presented for the KEGG pathway map of *E. coli*. Up-regulated fluxes are green, down regulated fluxes red, and fluxes without a regulation requirement are colored yellow. No-flux reactions and reactions without KEGG IDs are omitted.

First, we note that 306 of the 709 differentially expressed genes are present in the genome-scale metabolic model *i*ML1515 (Monk et al., 2017). In Figures 7, 8, we present the *E. coli* K-12 MG1655 KEGG (Kyoto Encyclopedia of Genes and Genomes) (Kanehisa, 2019; Kanehisa et al., 2019) pathway map. Colored in green and red are the up- and down-regulated reaction flux predictions based on the *i*ML1515 genome- scale metabolic model, respectively, with yellow representing non-zero reaction fluxes that are not required to be regulated.

In Table 4 we present the individual regulation pattern for ten different biocides (Pereira et al., 2020) as well as for Pd. We found that the general distribution of reactions into different categories is fairly consistent between biocides, with between 50 and 125 up-regulated reactions, 550 to 600 down-regulated reactions, and eight to 15 ambiguous reactions. There are approximately 2,000 reactions in the metabolic network that do not carry flux. The Pd flux distribution differs substantially from the others, with the majority of reactions (1,485 out of 2,712) being categorized as ambiguous, 1,090 not carrying flux, 17 up-regulated, and 120 down-regulated reactions.

**TABLE 4:** Differentially regulated flux predictions for the given condition.

	**BENZ**	**XID**	**PHE**	**GLUTA**	**H_2_O_2_**	**ETOH**	**ISOP**	**PERA**	**POV**	**SOD**	**PAL**
Up-regulation	122	72	80	48	103	84	79	76	71	55	17
Down-regulation	558	575	590	593	555	580	573	563	585	590	120
Ambiguous	12	14	9	9	11	13	15	10	8	11	1485

*The table shows differentially regulated flux predictions for multiple biocides employing the *E. coli* model iML1515. The listed chemicals are Benzalkonium chloride BENZ, Chlorhexidine XID, Chlorophene PHE, Glutaraldehyde GLUTA, Hydrogen peroxide H2O2, Ethanol ETOH, Isopropanol ISOP, Peracetic acid PERA, Povidone- iodine POV, Sodium hypochlorite SOD, and Palladium PAL (Pereira et al., 2020). Note that we used the transcriptomic response after twelve hours.*

Comparing the stress responses between the Pd stress environment and the biocide set as a whole (using the median flux values and upper and lower bounds of each reaction across biocides as a proxy for a generic response), we found the most significant commonality between the down-regulated sets (Table 5). Almost all reactions down-regulated in Pd (105 out of 120) are also being generically down-regulated under biocide stress conditions. However, we also found several major differences: in particular, ambiguous reactions are far more common in the Pd set, representing more than half (1,485 out of 2,712, or 55%) of all of the reactions. This is also reflected in Figures 7, 8, where we can see that the Pd condition is dominated by ambiguous fluxes, whereas biocide stress in general corresponds to consistently down-regulated fluxes.

**TABLE 5:** Similarity of stress responses.

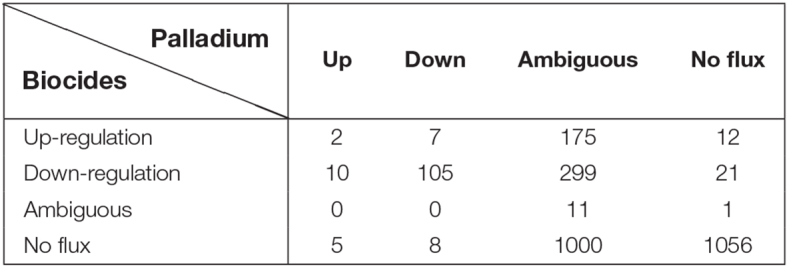

*The table shows the similarity of the predicted stress responses. We summarized the biocide stress response and compared it to the palladium stress response. Here, we list the number of reactions in the respective categories.*

## Discussion

The general and oxidative stress-related effects of Pd stress to *E. coli* were compared to published work on the transcriptomic response of *E. coli* to other heavy metal stresses, namely, Ni (Gault et al., 2016), Co (Fantino et al., 2010), Cu (Kershaw et al., 2005), Zn (Lee et al., 2005), Ag (McQuillan and Shaw, 2014), Hg (LaVoie and Summers, 2018), and Cd (Helbig et al., 2008; A. Wang and Crowley, 2005), and to the response of the *Enterobacteriaceae* bacterium LSJC7 to As stress (Zhang et al., 2016). Pd-induced oxidative stress effects were also compared to other non-heavy metal-related oxidative stresses, namely, Cl (Wang et al., 2009) and biocide (Pereira et al., 2020) stresses. We found that several Pd stress-related effects are similar to the effects of other heavy metal or oxidative stresses (discussed in the section on common transcriptional changes, below). Interestingly, Pd stress caused unique effects that were not reported in the aforementioned heavy metal and oxidative stress studies (discussed in uncommon transcriptional changes, below).

### Common Transcriptional Changes

This section discusses transcriptional changes that have been shown to occur in heavy metal stress responses previously. Overall, the challenge with Pd ions induced a multitude of expected changes in general stress response mechanisms and especially in efflux systems. A number of changes in basic energy metabolism were also observed in other heavy metal stress responses previously.

#### Protein Synthesis Arrest

Protein synthesis uses most of the cellular ATP (Pontes et al., 2015). The global down-regulation of the translation apparatus including the expression of the r-proteins, the elongation factors, and the tRNA processing genes suggests that the cells are trying to save energy for stress-related pathways rather than translation. This process of energy conservation was also found in other heavy metal stresses including Ni (Gault et al., 2016), Hg (LaVoie and Summers, 2018), Ag (McQuillan and Shaw, 2014) and Cd (Wang and Crowley, 2005).

#### RpoH and RpoS Sigma Factor Up-Regulation

When *E. coli* is exposed to high temperatures or any other condition that results in unfolded proteins, including exposure to heavy metals, the heat shock sigma factor, RpoH, is induced (Foster, 2005). RpoH regulates the expression of approximately 30 genes encoding heat shock response proteins. In contrast to this, the stationary phase and stress response sigma factor, RpoS, is triggered by other stress conditions that have the common property of arresting growth (Prabhakaran et al., 2016), such as oxidative stress (Eisenstark et al., 1996). Pd stress induced both sigma factors, this was also the case in Ag (McQuillan and Shaw, 2014) and Hg (LaVoie and Summers, 2018) stresses. Ni (Gault et al., 2016) and Cl (Wang et al., 2009) stresses induced only RpoS.

#### Heat Shock Response and Stress Proteins

Several heavy metals cause protein damage through cross-linking, disrupting their 3D structures and allosteric movements. The heat shock and stress-related proteolytic enzymes and chaperones help in the stabilization, re-folding, or the proteolysis of misfolded proteins. Five of the known heat shock and stress proteins were in top 20 highest up-regulated genes. The total number of up-regulated genes encoding heat shock and stress proteins due to Pd stress (28 genes) is very high compared to other heavy metal stresses. Ag stress (McQuillan and Shaw, 2014) comes in second where 17 genes were up-regulated. Other heavy metal stresses had less than 10 up-regulated genes, including Hg (LaVoie and Summers, 2018) and Cd (Helbig et al., 2008). Ni (Gault et al., 2016), Zn (Lee et al., 2005), Cu (Kershaw et al., 2005), Cl (Wang et al., 2009). Biocide stress (Pereira et al., 2020) induced only stress proteins and not heat shock proteins, based on RpoH and RpoS up-regulation explained in the previous section. This suggests that the damage caused by Pd on the protein level is even higher than by other heavy metals and requires a high number proteases and chaperones to deal with it.

#### Multi-Drug Efflux Systems

These systems are up-regulated in various stress conditions. The genes of these systems induce the expression of drug/toxin efflux system genes and limit passive uptake by decreasing porin expression (Alekshun and Levy, 1999). Pd stress induced the major three multiple drug efflux systems, MarRAB, MdtABC, and AcrAB. A similar effect was induced by Hg stress (LaVoie and Summers, 2018), while other heavy metal and oxidative stresses induced less than 3 efflux systems; 2 in case of Zn (Lee et al., 2005); 1 in case of Cd (Helbig et al., 2008) and Cl (Wang et al., 2009); and none in case of Ni (Gault et al., 2016), Ag (McQuillan and Shaw, 2014), As (Zhang et al., 2016), Co (Fantino et al., 2010), and Cu (Kershaw et al., 2005). This might suggest that Pd toxicity is highly unwanted for the cells or potentially the cells do not know what they are dealing with, and as a result, all the multi-drug efflux systems are up-regulated.

#### Oxidative Stress Response

The formation of reactive intermediates, mainly radicals that react with oxygen and produce reactive oxygen species (ROS) is a common effect of heavy metal stresses (metal-induced oxidative stress), as a result of changing the redox state of the cell and their ability to self-oxidation (Kappus, 1987). These ROS interact with thiols, metal centers, nucleotide bases and lipids (Fang, 2004), which results in damages to nucleic acids, proteins, and lipids, and therefore, disrupting the normal cellular function (Ray et al., 2012). The two oxidative stress response systems in *E. coli* were up-regulated due to Pd stress, OxyRS and SoxRS. These two systems modulate a cascade of more than 100 genes encoding proteins with diverse stress-related biological functions including superoxide scavenging, DNA and protein repair, recycling of macromolecules, xenobiotics efflux, carbon metabolism, and NADPH regeneration (Pomposiello and Demple, 2001; Blanchard et al., 2007). Pd stress induced 32 genes from these pathways, including the *soxS* regulator gene that was the 6th highest up-regulated gene, increasing 240-fold. Similar effects are found in other heavy metal stresses including Ag (McQuillan and Shaw, 2014), As (Zhang et al., 2016), Cd (Helbig et al., 2008), Cu (Kershaw et al., 2005), Hg (LaVoie and Summers, 2018), Ni (Gault et al., 2016), and Zn (Lee et al., 2005).

#### Fe-S Clusters Biogenesis

Many genes encode proteins that contain Fe-S clusters. These redox-active proteins have prominent roles in several important cellular processes, including respiration, central metabolism, and gene regulation (Y. Zhang et al., 2016). Fe-S proteins can by damaged by oxidative stress (Py and Barras, 2010). *E. coli* has two systems that are responsible for Fe-S clusters assembly and repair, the *isc* and *suf* operons (Outten et al., 2004). All genes of the *iscARSU* system were up-regulated due to Pd stress, together with the *nfuA* gene that is involved in Fe-S biogenesis under iron starvation conditions. This suggests that Pd-induced oxidative stress is damaging the Fe-S proteins and hence, the need to repair these proteins is increased. The *sufABC* operon was not modulated. Similar behavior was observed with As (Zhang et al., 2016), Cd (Helbig et al., 2008), Co (Fantino et al., 2010), and Zn (Lee et al., 2005) stresses. Copper stress (Kershaw et al., 2005) had the opposite effect, where the *suf* operon was up-regulated and not the *isc* operon. There are other heavy metal stresses that induced both *isc* and *suf* operons, including Ag (McQuillan and Shaw, 2014), Hg (LaVoie and Summers, 2018), and Cl (Wang et al., 2009) stresses. Ni stress (Gault et al., 2016) did not induce either of these operons.

#### Energy Production

In this study, *E. coli* was cultivated anaerobically, and accordingly, the cells are predicted to mainly use anaerobic respiration and fermentation for energy production. It is worth noting though that complete absence of oxygen cannot be guaranteed because closed tubes might still allow minimal diffusion of oxygen into the sample (see section “DISCUSSION”). The M63 minimal culture medium used to cultivate *E. coli* did not include any known amounts of terminal electron acceptors for anaerobic respiration (Figure 3G), except the minimal concentrations of HNO_3_ solvent added, this leaves *E. coli* with fermentation as the main energy production process.

The first step of energy production is the degradation of carbohydrates to make pyruvate (glycolysis). *E. coli* has three native pathways to make pyruvate from glucose (EMP, OPPP, and EDP). None of these pathways were heavily modulated due to Pd stress. Interestingly, the genes involved in making pyruvate from other sugars such as glycerol, L-fucose, D-glucuronate, D-galacturonate, D-galactonate, and D-galactarate were highly up-regulated (summarized in Figures 3B,C). None of these carbohydrates were included in the culture medium either, where the sole carbon source was glucose. Glycerol-3-P can be synthesized from deacetylated phospholipids in the periplasm by the enzyme GlpQ (periplasmic glycerophosphoryl diester phosphodiesterase). These deacetylated phospholipids are released from dying cells and imported through the outer membrane by FadL and through the inner membrane by FadD proteins. GlpQ, FadL, and FadD were all up-regulated upon Pd exposure. This suggests that *E. coli* is generating energy through glycerol metabolism simultaneously with glucose metabolism to cope with Pd stress. A similar effect was reported in the same *E. coli* strain when it was exposed to atmospheric pollution (Zhang T. et al., 2019) and heavy metal mixtures (Trchounian et al., 2016). Regarding the other aforementioned carbohydrates, we assume that the cells up-regulated the transport systems for 14 different sugars out of a desperate, general need for energy.

The second step is making energy from pyruvate through fermentation. *E. coli* has different fermentative pathways to generate energy (marked in blue color in Figure 3D). The fermentative pathways of D-lactate, succinate and hydrogen were up-regulated. The *hycA* gene encoding the transcriptional repressor of the formate regulon was the strongest down-regulated gene due to Pd stress, decreasing 520-fold. This regulon includes the genes *hycBCDEFGHI* of hydrogenlyase FHL (also called [Ni-Fe] hydrogenase 3), and the gene for formate dehydrogenase H, *fdhF* (Skibinski et al., 2002). These 2 enzymes form a formate hydrogenlyase complex, which is responsible for the vast majority of H_2_ production that occurs during fermentation in *E. coli* (McDowall et al., 2014). The bioreduction of Pd^2+^ ions requires the involvement of [Ni-Fe] hydrogenases. Deplanche et al. showed that a negligible Pd^2+^ ions reduction happens in an *E. coli* mutant strain genetically deprived of all hydrogenase activity (Deplanche et al., 2010). This might suggest that *E. coli* was detoxifying Pd ions through reduction in addition to the modulation of ion transporters.

Phosphoenolpyruvate, the starting molecule for the fermentation process, is also the precursor for aromatic amino acids synthesis together with erythrose-4-P. Aromatic amino acid synthesis pathways were massively down-regulated due to Pd stress (Figure 4F). This suggests that *E. coli* was trying to save phosphoenolpyruvate for energy production. In addition, the synthesis of acetyl-CoA, another pathway feeding into fermentation processes, though beta-oxidation cycle was up-regulated. This demonstrates that *E. coli* has modulated its gene expression for energy production over multiple pathways to cope with Pd stress (Figures 3A–E).

Interestingly, the anaerobic respiration genes were modulated too, despite the absence of terminal electron acceptors (Figure 3G). Upon Pd exposure, the genes encoding formate and glycerol-3-P dehydrogenases and hydrogenase 2 were up-regulated together with nitrate reductase, but glucose and malate dehydrogenases were down-regulated. The up-regulation of nitrate reductase might be due to signaling induced through the addition of minimal amounts of HNO_3_, and this could partly explain the modulation of the dehydrogenases. However, the up-regulation of nitrate reductase could also be a general stress effect, as a similar effect was reported in As stress (Zhang et al., 2016).

Similar to Fe-S clusters, the sulfur-containing amino acids, cysteine and methionine, are vulnerable to oxidation. Methionine residues are extremely sensitive to oxidative stress and they are easily oxidized to methionine sulfoxide (Liu et al., 2018). The oxidation of these amino acids makes them unavailable for metabolic processes (Drazic and Winter, 2014). *E. coli* has a dedicated system for methionine oxidation repair, methionine sulfoxide reductase YedYZ (or MsrPQ) that are expressed under oxidative stress conditions (Yin et al., 2015). This reductase takes electrons from the quinone pool and gives it to methionine sulfoxide as a terminal electron acceptor (Ezraty et al., 2005). The expression of these genes was vastly up-regulated due to Pd stress, both were in the top 20 up-regulated genes, with *yedY* increasing 96-fold and *yedZ* increasing 112-fold. A similar behavior was seen in *E. coli* (S. Wang et al., 2009), *Enterococcus faecalis* (Zhao et al., 2010), *Pseudomonas fluorescens* (Lipus et al., 2019), and *Staphylococcus aureus* (Singh and Singh, 2010) when they were exposed to oxidative stress. The up-regulation of methionine sulfoxide reductase might suggest that *E. coli* was using methionine sulfoxide also as a terminal electron acceptor for the anaerobic respiration as another way to generate energy, and this could also partly explain the modulation of the anaerobic respiration dehydrogenases.

For the respiratory quinone pool, the expression of ubiquinone was up-regulated but the expression of menaquinone and demethyl-menaquinone was down-regulated. Ubiquinone is known to be a universal electron acceptor, accepting electrons from all the dehydrogenases, while menaquinone and demethyl-menaquinone accept electrons only from hydrogen, formate, glycerol-3-P, and NADH dehydrogenases (Søballe and Poole, 1999). The biosynthesis of one universal quinone that serves all the dehydrogenases instead of biosynthesizing three, from an energy perspective, could be the reason for this modulation. All the modulated genes related to anaerobic respiration are summarized in Table 6.

**TABLE 6:** Summary of anaerobic respiration differentially expressed genes.

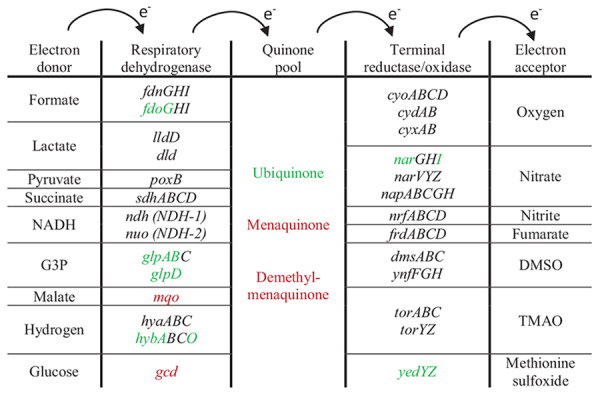

*The green color indicates up-regulation and the red color indicates down-regulation.*

#### Amino Acid Biosynthesis and Transport

Possibly due to methionine oxidation, the biosynthesis and uptake of methionine were heavily up-regulated in parallel to the up-regulation of methionine sulfoxide repair system. The biosynthesis and uptake of arginine and histidine was also highly up-regulated due to Pd stress. Arginine biosynthesis is elevated in nutrient limitation and stress conditions (Weerasinghe et al., 2006), and has been suggested to help bacteria in adaptation to oxidative stress (Tiwari et al., 2018; Barrientos-Moreno et al., 2019). The biosynthesis of histidine was also reported to be elevated in heavy metal stresses. Histidine helps the bacteria in the detoxification of metal ions through the sequestration and complexation of ions by histidine-rich peptides (Isarankura-Na-Ayudhya et al., 2018). Cysteine biosynthesis was not up-regulated due to Pd exposure despite being an easy target for oxidation. Interestingly, the same behavior was also found in HgCl_2_ stress (LaVoie and Summers, 2018) despite the fact that mercury has a strong affinity for thiol groups (Syversen and Kaur, 2012). Under HgCl_2_ exposure, the biosynthesis of methionine and histidine was up-regulated but the biosynthesis of cysteine was unchanged. The biosynthesis of most other amino acids was down-regulated or unchanged for Pd, HgCl_2_, Ni (Gault et al., 2016), Ag (McQuillan and Shaw, 2014) and, As (Zhang et al., 2016) stresses.

#### Inorganic Ion Transport Modulation

The most common bacterial strategies to deal with heavy metal stresses are the prevention of the metal ion from entering the cell and/or the active transport of the metal ion out of the cell (Nies and Silver, 2007). Both strategies were found to be applied by *E. coli* for Pd detoxification. For the first strategy, 8 metal ion import systems were down-regulated. The nickel-binding periplasmic protein NikA from the nickel transporter complex was down-regulated. Similar behavior was also found in Hg (LaVoie and Summers, 2018) and Co (Fantino et al., 2010) stresses. This shows that this system might be involved more generally in divalent heavy metal ion transport to the *E. coli*. For unknown reasons, this system was not down-regulated in Ni stress (Gault et al., 2016). Interestingly, all Fe^2+^ and Fe^3+^ import systems were down-regulated. This strongly suggests that Pd stress causes the disruption of Fe homeostasis and suggest that these systems could also be more directly involved in Pd transport to the cell. In oxidative stress conditions, iron import systems are usually up-regulated to take up more Fe for the biogenesis and repair of Fe-S clusters. Four of these systems were up-regulated in Ag stress (McQuillan and Shaw, 2014), while in As (Zhang et al., 2016), Cu (Kershaw et al., 2005) and biocide (Pereira et al., 2020) stresses, 3 or fewer systems were up-regulated. Other heavy metal stresses also caused Fe homeostatic disruption and therefore, some of the Fe import systems were down-regulated, including Co (Fantino et al., 2010), Cd (Helbig et al., 2008), Hg (LaVoie and Summers, 2018), and Ni (Gault et al., 2016) stresses. In addition to Fe import systems, the biosynthesis of enterobactin siderophore required for Fe^3+^ import was down-regulated, while the iron storage system was up-regulated. Similar findings were observed in Ni stress (Gault et al., 2016), where the cellular Fe content of Ni-treated *E. coli* was found to be less than the control. The zinc import system ZinT was up-regulated together with zinc resistance-associated protein ZraP (the latter is the 4th highest up-regulated, increasing 357-fold). This suggests that Pd might cause Zn homeostasis disruption too, supported by the fact that Zn homeostasis gene *zraP* was highly up-regulated. In addition, the magnesium import system MgtA was up-regulated. This up-regulation together with the down-regulation of potassium uptake protein TrkG could be based on the disturbance of cellular electrolyte balance caused by Pd stress, and as a result the cells try to maintain this balance by modulating these systems. The up-regulation of the osmotically inducible lipoprotein B, OsmB, supports this proposition. The magnesium import system MgtA was also up-regulated in Hg stress (LaVoie and Summers, 2018).

For the second strategy (the active transport of the metal ions out of the cell), 6 metal ion export systems were up-regulated. The nickel/cobalt efflux system RcnAB was up-regulated, comparable to up-regulation in Ni (Gault et al., 2016), Co (Fantino et al., 2010), and Hg (LaVoie and Summers, 2018) stresses. This suggests that this efflux system is involved more generally in divalent heavy metal ion detoxification in *E. coli*. The genes of the low-affinity ZntABR system were also up-regulated. This system is known to export ions with the following selectivity: Pb, Zn, Cd, Cu, Ni, and Co (Dutta et al., 2007). This system was up-regulated in Hg (LaVoie and Summers, 2018), Cd (Helbig et al., 2008), Co (Fantino et al., 2010), and Zn (Lee et al., 2005), but not in Ni (Gault et al., 2016) and Cu (Kershaw et al., 2005) stresses. This suggests that this system might also bind Pd ions, but this has to be confirmed. The copper-exporting ATPase CopA and the copper oxidase CueO were also up-regulated. These genes are involved in copper detoxification through efflux and through oxidizing Cu^+^ to Cu^2+^, respectively, and are up-regulated in many heavy metal stress situations (Grass and Rensing, 2001). This common heavy metal effect might be explained as an attempt to detoxify heavy metals by oxidation. It might also be that heavy metal exposure disrupts Cu homeostasis. Interestingly, Ni (Gault et al., 2016), Zn (Lee et al., 2005), Cu (Kershaw et al., 2005), and Ag (McQuillan and Shaw, 2014) stresses induced the *cus*ABC genes encoding a copper export system, but Pd exposure did not. This might suggest that Pd does not cause a disruption of Cu homeostasis as strongly as Fe and Zn. The arsenical resistance system ArsBCR was heavily up-regulated, with two of these genes in the top 20 up-regulated genes. This system was also up-regulated in As (Zhang et al., 2016), Hg (LaVoie and Summers, 2018), and Cd (Helbig et al., 2008) stresses. Moreover, the Ca^2+^/H^+^ antiporter ChaA and Na^+^/H^+^ antiporter NhaABR were both up-regulated. Similar behavior was found in Hg stress (LaVoie and Summers, 2018). The reason of this modulation could be the cellular attempt to restore the pH balance disturbed by Pd stress. Finally, the potassium efflux system KefCF system was down-regulated, which could be implicated in electrolyte and osmotic balancing together with TrkG and MgtA discussed above.

#### Down-Regulation of Motility and Biofilm Structures

Flagellar motility, fimbriae, and curli fibers are energetically costly structures (LaVoie and Summers, 2018). Like with protein synthesis arrest, Pd stress resulted in the down-regulation of these structures that are maybe less critical to immediate survival. For flagellar proteins, 8 genes were down-regulated upon Pd exposure, similar down-regulation was also found in Ni (Gault et al., 2016), Hg (LaVoie and Summers, 2018), Cu (Kershaw et al., 2005) and biocide (Pereira et al., 2020) stresses. The genes encoding biofilm structures suffered from a huge down-regulation too, the list includes the *yhcA* gene required for fimbriae biogenesis that was the 3rd strongest down-regulated gene, decreasing 197-fold, and the *csgE* gene required for curli production and assembly that was the 2nd strongest down-regulated gene, decreasing 338-fold. For biofilm synthesis, Cu (Kershaw et al., 2005) and Zn (Lee et al., 2005) stresses resulted in the down-regulation of the genes encoding fimbriae and curli proteins, but Ni (Gault et al., 2016), Hg (LaVoie and Summers, 2018), Cl (Wang et al., 2009), and biocide (Pereira et al., 2020) stresses induced them.

### Uncommon Transcriptional Changes

This section discusses transcriptional changes that have not previously been observed or discussed in other heavy metal stresses, and that might be specific to Pd stress. It also discusses changes that are counterintuitive or opposite to what has been described previously for other heavy metal stress conditions.

#### Down-Regulation of DNA Repair Genes

Heavy metal and oxidative stresses cause damage to nucleic acids through ROS. An up-regulation in DNA repair genes was found in Hg (LaVoie and Summers, 2018), Cd (Helbig et al., 2008), Zn (Lee et al., 2005), As (Y. Zhang et al., 2016), Cl (Wang et al., 2009), and biocide (Pereira et al., 2020) stresses. DNA repair genes were unchanged in Ni (Gault et al., 2016), Cu (Kershaw et al., 2005), or Co (Fantino et al., 2010) stresses. Pd exposure, surprisingly, caused a down-regulation of DNA repair genes.

#### Nucleotide Transport and Metabolism

Purine and pyrimidine transport and metabolism genes were severely down-regulated due to Pd stress. All the permeases responsible for nucleotide import were down-regulated. The genes involved in the biosynthesis of nucleotides through both the *de novo* synthesis and salvage pathways were down-regulated. At the same time, the metabolic pathways for nucleotide hydrolysis were highly up-regulated, including the *hiuH* gene that was the 2nd highest up-regulated gene, increasing 1465-fold. Interestingly, Ni (Gault et al., 2016) and Cd (Pan et al., 2017) stresses included a similar effect, but an opposite effect was seen in *Desulfovibrio vulgaris* when it was exposed to oxidative stress (Zhang et al., 2006). Nevertheless, this is not a common heavy metal or oxidative stress-related effect. Some microorganisms, e.g., *Klebsiella pneumoniae* can utilize purines as carbon or nitrogen source (Tyler, 1978). *E. coli* can utilize purines as nitrogen source (Xi et al., 2000). This major down-regulation in nucleotide metabolism might suggest that *E. coli* is using nucleotides as nitrogen source instead of using them as building blocks for DNA and RNA. This could also explain the down-regulation of DNA repair genes (see section above).

#### Coenzyme Transport and Metabolism

Palladium stress resulted in the down-regulation of several genes related to the biosynthesis and transport of vitamins, including vitamins B1, B2, B3, B6, B9, and B12. A modulation in the genes of any of these or other vitamins to the best of our knowledge was not reported before in any heavy metal or oxidative stress study. The reason for such modulation remains unclear.

#### Cell Division and Cell Wall Biogenesis

Some cell cycle control, cell division, and cell wall biogenesis genes were down-regulated due to Pd stress. Many of these genes were related to LPS biosynthesis. This effect might be explained by the general poor health status of the bacterial cells, that prefer to shut down these genes in order to conserve energy for stress related pathways. LPS also has high affinity to divalent metallic ions (Ferris and Beveridge, 1986; Kotrba et al., 1999), and the down-regulation of LPS biosynthesis genes might also contribute to a reduction in Pd binding and uptake.

#### Sulfur Transport and Metabolism

The genes of the transporter complex of sulfate/thiosulfate, *cysAWTP*, and the hydrogen sulfide biosynthesis genes, *cysCDHIJN*, are differentially expressed in heavy metal and oxidative stresses. Some of these genes were up-regulated in Ag (McQuillan and Shaw, 2014), Cd (Helbig et al., 2008), Zn (Lee et al., 2005), and Cl (S. Wang et al., 2009) stresses, and down-regulated in Hg (LaVoie and Summers, 2018), Ni (Gault et al., 2016), and Co (Fantino et al., 2010) stresses. None of these genes were modulated due to Pd stress. This might show that Pd does not affect sulfur homeostasis in *E. coli*, unlike the situation with Fe, Cu and Zn.

#### Carbohydrate Transport

Palladium stress induced the up-regulation of 14 different sugar transport systems. The uptake of maltose, galactose, trehalose, fructose, gluconate, galactofuranose, sorbitol, mannose, galactarate, L-fucose, xylose, ribose, L-arabinose, and glycerol was up-regulated. Such a systematic up-regulation was not found as an effect to any heavy metal stress before, in fact, the heavy metal and oxidative stress studies used in this paper did not record an up-regulation of any single sugar transport system. On the contrary, in Hg stress (LaVoie and Summers, 2018), the expression of several carbohydrate transport genes was down-regulated. The reasons for this remain unclear, but we speculate that under the anaerobic conditions approximated in our experiments, *E. coli* markedly tries to take up different possible fermentation substrates for energy, despite the fact that these sugars are not present in the medium.

### Genome-Scale Metabolic Modeling

Our RNA-Seq data was used in genome-scale metabolic modeling and the results that represent changes in metabolic pathways and fluxes were compared to our (manual) analysis of up- and down-regulation of individual genes and operons. The genome-scale analysis was also compared to a dataset where 10 different biocides that were analyzed under comparable experimental conditions (Pereira et al., 2020). This was done to narrow down what of the observed responses under Pd stress are general stress responses to a toxic substance, and what effects are indeed specific to Pd stress.

As shown in Figure 7 and Table 4, most of the metabolic reactions are either not affected (not shown in Figure 7) or down-regulated overall. Figure 8 shows that the Pd stress response does not require most reactions to be differentially regulated for an optimal match with the experimental expression data.

Note however, that the *i*ML1515 metabolic model only contains a subset of the experimentally determined differentially expressed genes. Nevertheless, several similarities in down-regulation were found in the pathways of cofactor and vitamin metabolism (highlighted in pink in Figures 7, 8), carbohydrate metabolism (highlighted in blue), and terpenoid metabolism (highlighted in green) to mention a few.

Especially for Pd stress response, many reactions are classified as ambiguous in our calculations. While it might be tempting to presume that this difference is a result of comparing a median (across multiple biocides) to a single value, we also see that the distribution of reactions in each of the individual biocide environments is generally similar to that of the median, and widely different from the Pd response. It seems more plausible that the difference in observed regulation is influenced by the environmental differences between the starting point for the biocide and Pd experiments. One such difference relates to available carbon and resulting capability of the cell to grow in the unstressed state. In the biocide experiments, the unstressed cell is able to allocate much of its input to biomass growth, which requires the production of a varied set of metabolites in fixed proportions. Any reduction in growth due to a limited set of bottlenecks would also reduce the necessary production of other metabolites. In contrast, the Pd environment has no external carbon source, and metabolism is simply directed toward survival, which from a model point of view primarily involves producing a sufficient amount of ATP, using whatever carbon source is available. If the cell has a wider set of alternative states available to meet such a requirement, this would lead to a greater uncertainty in possible flux distributions.

The conditions between Pd and biocides are also different as the latter are grown in an aerobic environment for 12 h in a defined medium. Hence, the organism has likely achieved a degree of adaptation to the new environment containing the stressor, possibly resulting in a lower growth rate (Pereira et al., 2020). In contrast, for Pd stress with short exposure time, the immediate stress response to the heavy metal is assessed. This results in many genes in comparison not being as significantly regulated based on the experimental data. For the biocides however, possibly due to the longer adaptation time, more genes are differentially expressed, resulting in an increased overlap with the model. Thus, the gx-FBA methodology is more likely to generate results of higher quality and result in a clearer pattern of up- and down- regulated reactions in comparison to the Pd stress response.

In summary, we observe that the biocide and Pd stress responses share certain commonalities, in particular with regards to a significant amount of down-regulated reactions; we also see a substantial difference evidenced by the large amount of reactions that are down-regulated under biocide stress conditions, but ambiguous under Pd stress, which may in part be a result of the differences in environmental conditions.

## Conclusion

The introduction of Pd stress to *E. coli* resulted in common heavy metal and oxidative stress effects as well as effects that seem to be unique for Pd exposure. The commons effects were: energy conservation through protein synthesis arrest and the down-regulation of motility and biofilm structures; cellular detoxication through the up-regulation of multi-drug efflux systems and inorganic ion transport complexes; the stabilization, re-folding, and degradation of misfolded proteins through up-regulation of heat-shock and stress sigma factors and proteins; the induction of OxyRS and SoxRS oxidative stress response systems; and the up-regulation of energy production and conversion pathways. Pd^2+^ has a standard reduction potential of + 0.95 V, which is higher than all the other heavy metal ions used for the comparison in this study (Zn^2+^ is −0.76 V, Cd^2+^ is −0.40 V, Co^2+^ is −0.28 V, Ni^2+^ is −0.26 V, As^3+^ is −0.22 V, Cu^2+^ is + 0.34 V, Ag^+^ is 0.80 V, and Hg^2+^ is + 0.85 V) (Mayer and Holze, 2001). The higher the reduction potential, the easier it is for the metal ions to get reduced and therefore, the higher the oxidative stress caused. This might explain the high oxidative stress levels that resulted from Pd exposure, compared to other heavy metals.

Interestingly, Pd stress resulted in some unique effects, namely, the down-regulation of DNA repair genes, massive down-regulation of nucleotide transport and metabolism genes, down-regulation of coenzyme transport and metabolism genes especially for the B vitamins, down-regulation of cell wall biogenesis genes, and a massive up-regulation of carbohydrate transport genes. We previously observed that Pd stress might cause *E. coli* to go into viable but non-culturable (VBNC) state (data not shown). In this state, the cells are metabolically active (confirmed by colorimetric assays and viability assay kits) but unable to grow on agar plates. The massive down-regulation of nucleotide transport and metabolism, coenzyme transport and metabolism, and cell wall biogenesis genes might explain the reason behind *E. coli* going into this state.

In addition, Pd stress was found to disrupt the homeostasis of iron, copper, and zinc. Furthermore, the cells were found to detoxify Pd ions through four different strategies: the prevention of metal ions from entering the cells through the down-regulation of several inorganic ion transporter complexes, the active transport of the metal ions out of the cell through the up-regulation of multi-drug efflux systems and inorganic ion transporter complexes, the enzymatic alteration of the metal ions to a less toxic form (Pd nanoparticles) through the up-regulation of hydrogenases (Deplanche et al., 2010), and the potential intracellular sequestration of the metal ions by binding to proteins such as histidine-rich peptides through the up-regulation of histidine transport and biosynthesis genes. Pd is not a trace metal relevant to the growth of *E. coli*, and it seems improbable that the bacteria harbor sensory systems that would directly respond to the presence of Pd ions. More likely, the observed responses and changes in gene expression were mainly caused by the secondary effects of general heavy metal toxicity. It is interesting to note that *E. coli* shows some responses that seem to be specific for Pd, and further research is needed to elucidate the underlying signaling processes. The results presented here are important for efforts to use bacteria in bioremediation of heavy metal wastes (Foulkes et al., 2016; Murray et al., 2020), or to produce metal nanoparticles. Engineering strains for such purposes is only possible based on a thorough understanding of the physiological processes induced by the presence of Pd and other heavy metal ions, both on a single-gene and a systems level.

## Data Availability Statement

The data that supports the findings of this study are available in the Supplementary Material of this article. The raw data is publicly available on ArrayExpress database, https://www.ebi.ac.uk/arrayexpress/experiments/E-MTAB-10803/.

## Author Contributions

NJ performed experiments, analyzed data, and wrote the manuscript. AS, CS, and AV analyzed data and edited the manuscript. EA acquired funding, supervised data analysis, and edited the manuscript. DL acquired funding, supervised experiments and data analysis, and edited the manuscript. All authors contributed to the article and approved the submitted version.

## Conflict of Interest

The authors declare that the research was conducted in the absence of any commercial or financial relationships that could be construed as a potential conflict of interest.

## Publisher’s Note

All claims expressed in this article are solely those of the authors and do not necessarily represent those of their affiliated organizations, or those of the publisher, the editors and the reviewers. Any product that may be evaluated in this article, or claim that may be made by its manufacturer, is not guaranteed or endorsed by the publisher.

## Footnotes

1
https://www.macrotrends.net/2542/palladium-prices-historical-chart-data

2
https://www.ncbi.nlm.nih.gov/research/cog

3
https://www.uniprot.org/

4
https://www.ebi.ac.uk/QuickGO/

5
https://biocyc.org/

## References

[B1] Alekshun M. N. Levy S. B. (1999). The mar regulon: multiple resistance to antibiotics and other toxic chemicals. *Trends Microbiol.* 7 410–413. 10.1016/S0966-842X(99)01589-9 10498949

[B2] Altuvia S. Zhang A. Argaman L. Tiwari A. Storz G. (1998). The *Escherichia coli* oxyS regulatory RNA represses fhlA translation by blocking ribosome binding. *EMBO J.* 17 6069–6075. 10.1093/emboj/17.20.6069 9774350 PMC1170933

[B3] Alva A. Sabido-Ramos A. Escalante A. Bolívar F. (2020). New insights into transport capability of sugars and its impact on growth from novel mutants of *Escherichia coli*. *Appl. Microbiol. Biotechnol.* 104 1463–1479. 10.1007/s00253-019-10335-x 31900563

[B4] Baba T. Ara T. Hasegawa M. Takai Y. Okumura Y. Baba M. (2006). Construction of *Escherichia coli* K-12 in-frame, single-gene knockout mutants: the keio collection. *Mol. Syst. Biol.* 2:2006.0008. 10.1038/msb4100050 16738554 PMC1681482

[B5] Barrientos-Moreno L. Molina-Henares M. A. Pastor-García M. Ramos-González M. I. Espinosa-Urgel M. (2019). Arginine biosynthesis modulates pyoverdine production and release in *Pseudomonas* putida as part of the mechanism of adaptation to oxidative stress. *J. Bacteriol.* 201:e00454. 10.1128/JB.00454-19 PMC6805116 31451546

[B6] Baxter-Plant V. S. Mabbett A. N. Macaskie L. E. (2002). Bacteria, their precious metal armour, and a new weapon against waste. *Microbiol. Today* 29 80–81.

[B7] Biffis A. Centomo P. Del Zotto A. Zecca M. (2018). Pd metal catalysts for cross-couplings and related reactions in the 21st century: a critical review. *Chem. Rev.* 118 2249–2295. 10.1021/acs.chemrev.7b00443 29460627

[B8] Blanchard J. L. Wholey W. Y. Conlon E. M. Pomposiello P. J. (2007). Rapid changes in gene expression dynamics in response to superoxide reveal SoxRS-dependent and independent transcriptional networks. *PLoS One* 2:e1186. 10.1371/journal.pone.0001186 18000553 PMC2064960

[B9] Bruins M. R. Kapil S. Oehme F. W. (2000). Microbial resistance to metals in the environment. *Ecotoxicol. Environ. Saf.* 45 198–207. 10.1006/eesa.1999.1860 10702338

[B10] Cheng H. Y. Soo V. W. C. Islam S. McAnulty M. J. Benedik M. J. Wood T. K. (2014). Toxin GhoT of the GhoT/GhoS toxin/antitoxin system damages the cell membrane to reduce adenosine triphosphate and to reduce growth under stress. *Environ. Microbiol.* 16 1741–1754. 10.1111/1462-2920.12373 24373067

[B11] Choi H. J. Kim S. J. Mukhopadhyay P. Cho S. Woo J. R. Storz G. (2001). Structural basis of the redox switch in the OxyR transcription factor. *Cell* 105 103–113. 10.1016/S0092-8674(01)00300-2 11301006

[B12] Cui Z. Bai X. Liu Y. Li S. (2017). Synthesis of palladium concave nanocubes with high−index facets and their catalytic properties. *Appl. Organometallic Chem.* 31:e3887. 10.1002/aoc.3887

[B13] De Corte S. Hennebel T. De Gusseme B. Verstraete W. Boon N. (2012). Bio−palladium: from metal recovery to catalytic applications. *Microb. Biotechnol.* 5 5–17. 10.1111/j.1751-7915.2011.00265.x 21554561 PMC3815268

[B14] De Windt W. Aelterman P. Verstraete W. (2005). Bioreductive deposition of palladium (0) nanoparticles on Shewanella oneidensis with catalytic activity towards reductive dechlorination of polychlorinated biphenyls. *Environ. Microbiol.* 7 314–325. 10.1111/j.1462-2920.2004.00696.x 15683392

[B15] Deplanche K. Caldelari I. Mikheenko I. P. Sargent F. Macaskie L. E. (2010). Involvement of hydrogenases in the formation of highly catalytic Pd(0) nanoparticles by bioreduction of Pd(II) using *Escherichia coli* mutant strains. *Microbiology* 156 2630–2640. 10.1099/mic.0.036681-0 20542928

[B16] Drazic A. Winter J. (2014). The physiological role of reversible methionine oxidation. *Biochim. Biophys. Acta* 1844 1367–1382. 10.1016/j.bbapap.2014.01.001 24418392

[B17] Du L. Jiang H. Liu X. Wang E. (2007). Biosynthesis of gold nanoparticles assisted by *Escherichia coli* DH5α and its application on direct electrochemistry of hemoglobin. *Electrochem. Commun.* 9 1165–1170. 10.1016/j.elecom.2007.01.007

[B18] Duffus J. H. (2002). “Heavy metals” a meaningless term?(IUPAC Technical Report). *Pure Appl. Chem.* 74 793–807. 10.1351/pac200274050793

[B19] Dutta S. J. Liu J. Stemmler A. J. Mitra B. (2007). Conservative and nonconservative mutations of the transmembrane CPC motif in ZntA: effect on metal selectivity and activity. *Biochemistry* 46 3692–3703. 10.1021/bi0616394 17326661

[B20] Eisenstark A. Calcutt M. J. Becker-Hapak M. Ivanova A. (1996). Role of *Escherichia coli* rpoS and associated genes in defense against oxidative damage. *Free Radic. Biol. Med.* 21 975–993. 10.1016/S0891-5849(96)00154-2 8937883

[B21] Ezraty B. Aussel L. Barras F. (2005). Methionine sulfoxide reductases in prokaryotes. *Biochim. Biophys. Acta* 1703 221–229. 10.1016/j.bbapap.2004.08.017 15680230

[B22] Fang F. C. (2004). Antimicrobial reactive oxygen and nitrogen species: concepts and controversies. *Nat. Rev. Microbiol.* 2 820–832. 10.1038/nrmicro1004 15378046

[B23] Fantino J. R. Py B. Fontecave M. Barras F. (2010). A genetic analysis of the response of *Escherichia coli* to cobalt stress. *Environ. Microbiol.* 12 2846–2857. 10.1111/j.1462-2920.2010.02265.x 20545747

[B24] Feliciano-Ramos I. Casañas-Montes B. García-Maldonado M. M. Menéndez C. L. Mayol A. R. Díaz-Vázquez L. M. (2014). Assembly of a cost-effective anode using palladium nanoparticles for alkaline fuel cell applications. *J. Chem. Educ.* 92 360–363. 10.1021/ed500230y 25691801 PMC4325606

[B25] Ferris F. G. Beveridge T. J. (1986). Site specificity of metallic ion binding in *Escherichia coli* K-12 lipopolysaccharide. *Can. J. Microbiol.* 32 52–55. 10.1139/m86-010 3516350

[B26] Foster P. L. (2005). Stress responses and genetic variation in bacteria. *Mutat. Res.* 569 3–11. 10.1016/j.mrfmmm.2004.07.017 15603749 PMC2729700

[B27] Foulkes J. M. Deplanche K. Sargent F. Macaskie L. E. Lloyd J. R. (2016). A novel aerobic mechanism for reductive palladium biomineralization and recovery by *escherichia coli*. *Geomicrobiol. J.* 33 230–236. 10.1080/01490451.2015.1069911

[B28] Fozo E. M. Kawano M. Fontaine F. Kaya Y. Mendieta K. S. Jones K. L. (2008). Repression of small toxic protein synthesis by the Sib and OhsC small RNAs (Molecular Microbiology (2008) 70, (1076-1093)). *Mol. Microbiol.* 70:1305. 10.1111/j.1365-2958.2008.06529.x PMC2597788 18710431

[B29] Gault M. Effantin G. Rodrigue A. (2016). Ni exposure impacts the pool of free Fe and modifies DNA supercoiling via metal-induced oxidative stress in *Escherichia coli* K-12. *Free Radic. Biol. Med.* 97 351–361. 10.1016/j.freeradbiomed.2016.06.030 27375130

[B30] Gavia D. J. Shon Y.-S. (2015). Catalytic properties of unsupported palladium nanoparticle surfaces capped with small organic ligands. *ChemCatChem* 7:892. 10.1002/cctc.201402865 25937846 PMC4415887

[B31] Gehring A. M. Bradley K. A. Walsh C. T. (1997). Enterobactin biosynthesis in *escherichia coli*: Isochorismate lyase (EntB) is a bifunctional enzyme that is phosphopantetheinylated by EntD and then acylated by ente using ATP and 2,3-dihydroxybenzoate. *Biochemistry* 36 8495–8503. 10.1021/bi970453p 9214294

[B32] Georgopoulos C. Welch W. J. (1993). Role of the major heat shock proteins as molecular chaperones. *Annu. Rev. Cell Biol.* 9 601–634. 10.1146/annurev.cb.09.110193.003125 8280473

[B33] Grass G. Rensing C. (2001). Genes involved in copper homeostasis in *Escherichia coli*. *J. Bacteriol.* 183 2145–2147. 10.1128/JB.183.6.2145-2147.2001 11222619 PMC95116

[B34] Gurobi Optimization (2014). *Gurobi Optimizer Reference Manual, 2015.* Available online at: http://www.gurobi.com.

[B35] Gurunathan S. Kalishwaralal K. Vaidyanathan R. Venkataraman D. Pandian S. R. K. Muniyandi J. (2009). Biosynthesis, purification and characterization of silver nanoparticles using *Escherichia coli*. *Colloids Surf. B Biointerfaces* 74 328–335. 10.1016/j.colsurfb.2009.07.048 19716685

[B36] Heirendt L. Arreckx S. Pfau T. Mendoza S. N. Richelle A. Heinken A. (2019). Creation and analysis of biochemical constraint-based models using the COBRA Toolbox v.3.0. *Nat. Protoc.* 14 639–702. 10.1038/s41596-018-0098-2 30787451 PMC6635304

[B37] Helbig K. Grosse C. Nies D. H. (2008). Cadmium toxicity in glutathione mutants of *Escherichia coli*. *J. Bacteriol.* 190 5439–5454. 10.1128/JB.00272-08 18539742 PMC2493267

[B38] Hennebel T. Van Nevel S. Verschuere S. De Corte S. De Gusseme B. Cuvelier C. (2011). Palladium nanoparticles produced by fermentatively cultivated bacteria as catalyst for diatrizoate removal with biogenic hydrogen. *Appl. Microbiol. Biotechnol.* 91 1435–1445. 10.1007/s00253-011-3329-9 21590286

[B39] Hollinshead W. D. Rodriguez S. Martin H. G. Wang G. Baidoo E. E. K. Sale K. L. (2016). Examining *Escherichia coli* glycolytic pathways, catabolite repression, and metabolite channeling using Δ pfk mutants. *Biotechnol. Biofuels* 9:212. 10.1186/s13068-016-0630-y PMC5057261 27766116

[B40] Isarankura-Na-Ayudhya P. Thippakorn C. Pannengpetch S. Roytrakul S. Isarankura-Na-Ayudhya C. Bunmee N. (2018). Metal complexation by histidine-rich peptides confers protective roles against cadmium stress in *Escherichia coli* as revealed by proteomics analysis. *PeerJ* 6:e5245. 10.7717/peerj.5245 PMC6064632 30065864

[B41] Järup L. (2003). Hazards of heavy metal contamination. *Br. Med. Bull.* 68 167–182. 10.1093/bmb/ldg032 14757716

[B42] Jbara M. Maity S. K. Brik A. (2017). Palladium in the chemical synthesis and modification of proteins. *Angew. Chem. Int. Ed.* 56 10644–10655. 10.1002/anie.201702370 28383786

[B43] Kanehisa M. (2019). Toward understanding the origin and evolution of cellular organisms. *Protein Sci.* 28 1947–1951. 10.1002/pro.3715 31441146 PMC6798127

[B44] Kanehisa M. Sato Y. Furumichi M. Morishima K. Tanabe M. (2019). New approach for understanding genome variations in KEGG. *Nucleic Acids Res.* 47 D590–D595. 10.1093/nar/gky962 30321428 PMC6324070

[B45] Kappus H. (1987). Oxidative stress in chemical toxicity. *Arch. Toxicol.* 60 144–149. 10.1007/BF00296968 3304204

[B46] Karian H. (2003). *Handbook of Polypropylene and Polypropylene Composites, Revised and Expanded.* Boca Raton, FL: CRC Press.

[B47] Kershaw C. J. Brown N. L. Constantinidou C. Patel M. D. Hobman J. L. (2005). The expression profile of *Escherichia coli* K-12 in response to minimal, optimal and excess copper concentrations. *Microbiology* 151 1187–1198. 10.1099/mic.0.27650-0 15817786

[B48] Kotrba P. Dolečková L. De Lorenzo V. Ruml T. (1999). Enhanced bioaccumulation of heavy metal ions by bacterial cells due to surface display of short metal binding peptides. *Appl. Environ. Microbiol.* 65 1092–1098. 10.1128/aem.65.3.1092-1098.1999 10049868 PMC91149

[B49] Kundu S. Kalees Warran P. Mursalin S. M. Narjinary M. (2015). Synergistic effect of Pd and Sb incorporation on ethanol vapour detection of La doped tin oxide sensor. *J. Materi. Sci. Mater. Electron.* 26 9865–9872. 10.1007/s10854-015-3662-3

[B50] LaVoie S. P. Summers A. O. (2018). Correction: transcriptional responses of *Escherichia coli* during recovery from inorganic or organic mercury exposure. *BMC Genomics* 19:268. 10.1186/s12864-018-4631-z 29338696 PMC5769350

[B51] Lee L. J. Barrett J. A. Poole R. K. (2005). Genome-wide transcriptional response of chemostat-cultured *Escherichia coli* to zinc. *J. Bacteriol.* 187 1124–1134. 10.1128/JB.187.3.1124-1134.2005 15659689 PMC545701

[B52] Li H. (2013). Aligning sequence reads, clone sequences and assembly contigs with BWA-MEM. *ArXiv* [Preprint] Available online at: http://arxiv.org/abs/1303.3997. ArXiv:1303.3997

[B53] Liao Y. Smyth G. K. Shi W. (2014). FeatureCounts: an efficient general purpose program for assigning sequence reads to genomic features. *Bioinformatics* 30 923–930. 10.1093/bioinformatics/btt656 24227677

[B54] Lipus D. Vikram A. Gulliver D. Bibby K. (2019). Upregulation of peroxide scavenging enzymes and multidrug efflux proteins highlight an active sodium hypochlorite response in *Pseudomonas* fluorescens biofilms. *Biofouling* 35 329–339. 10.1080/08927014.2019.1605357 31066290

[B55] Liu G. Huang Y. Zhai L. (2018). Impact of nutritional and environmental factors on inflammation, oxidative stress, and the microbiome. *Biomed. Res. Int.* 2018:5606845. 10.1155/2018/5606845 PMC6091408 30151385

[B56] Lloyd J. R. Coker V. S. Kimber R. L. Pearce C. I. Watts M. P. Omajali J. B. (2020). New frontiers in metallic bio-nanoparticle catalysis and green products from remediation processes. *RSC Green Chem.* 2020 244–265. 10.1039/9781788016353-00244

[B57] Lloyd J. R. Yong P. Macaskie L. E. (1998). Enzymatic recovery of elemental palladium by using sulfate-reducing bacteria. *Appl. Environ. Microbiol.* 64 4607–4609. 10.1128/aem.64.11.4607-4609.1998 9797331 PMC106693

[B58] Lozet J. Mathieu C. (1993). “Dictionary of soil science,” in *Soil Science*, eds Lozet J. Mathieu C. (Paris: Technique et Documentation-Lavoisier).

[B59] Matsumoto T. Kamino M. Yamada R. Konishi Y. Ogino H. (2020). Identification of genes responsible for reducing palladium ion in *Escherichia coli*. *J, Biotechnol*, 324 7–10. 10.1016/j.jbiotec.2020.09.015 32971180

[B60] Mayer P. Holze R. (2001). Electrocatalysis of redox reactions by metal nanoparticles on graphite electrodes. *J. Solid State Electrochem.* 5 402–411. 10.1007/s100080000169

[B61] McDowall J. S. Murphy B. J. Haumann M. Palmer T. Armstrong F. A. Sargent F. (2014). Bacterial formate hydrogenlyase complex. *Proc. Natl. Acad. Sci. U.S.A.* 111 E3948–E3956. 10.1073/pnas.1407927111 25157147 PMC4183296

[B62] McQuillan J. S. Shaw A. M. (2014). Differential gene regulation in the Ag nanoparticle and Ag +-induced silver stress response in *Escherichia coli*: a full transcriptomic profile. *Nanotoxicology* 8(Suppl. 1) 177–184. 10.3109/17435390.2013.870243 24392705

[B63] Miller J. Schuler C. Ward J. Wylie F. Frea T. Delbene R. (1992). A short course. *Bact. Genet.* 37 509–509.

[B64] Miller M. A. Askevold B. Mikula H. Kohler R. H. Pirovich D. Weissleder R. (2017). Nano-palladium is a cellular catalyst for in vivo chemistry. *Nat. Commun.* 8:15906. 10.1038/ncomms15906 28699627 PMC5510178

[B65] Monk J. M. Lloyd C. J. Brunk E. Mih N. Sastry A. King Z. (2017). iML1515, a knowledgebase that computes *Escherichia coli* traits. *Nat. Biotechnol.* 35 904–908. 10.1038/nbt.3956 29020004 PMC6521705

[B66] Murray A. J. Mikheenko I. P. Deplanche K. Omajali J. B. Gomez-Bolivar J. Merroun M. L. (2020). Chapter 9: biorefining of metallic wastes into new nanomaterials for green chemistry, environment and energy. *RSC Green Chem.* 63 213–243. 10.1039/9781788016353-00213

[B67] Navid A. Almaas E. (2012). Genome-level transcription data of yersinia pestis analyzed with a new metabolic constraint-based approach. *BMC Syst. Biol.* 6:150. 10.1186/1752-0509-6-150 23216785 PMC3572438

[B68] Nies D. H. (1999). Microbial heavy-metal resistance. *Appl. Microbiol. Biotechnol.* 51 730–750. 10.1007/s002530051457 10422221

[B69] Nies D. H. Silver S. (2007). “Molecular microbiology of heavy metals,” in *Molecular Microbiology of Heavy Metals*, eds Nies D. H. Silver S. (Berlin: Springer Science & Business Media), 460.

[B70] Outten F. W. Djaman O. Storz G. (2004). A suf operon requirement for Fe-S cluster assembly during iron starvation in *Escherichia coli*. *Mol. Microbiol.* 52 861–872. 10.1111/j.1365-2958.2004.04025.x 15101990

[B71] Pan J. Huang X. Li Y. Li M. Yao N. Zhou Z. (2017). Zinc protects against cadmium-induced toxicity by regulating oxidative stress, ions homeostasis and protein synthesis. *Chemosphere* 188 265–273. 10.1016/j.chemosphere.2017.08.106 28886561

[B72] Pedersen K. Gerdes K. (1999). Multiple hok genes on the chromosome of *Escherichia coli*. *Mol. Microbiol.* 32 1090–1102. 10.1046/j.1365-2958.1999.01431.x 10361310

[B73] Pereira B. M. P. Wang X. Tagkopoulos I. (2020). Short-and long-term transcriptomic responses of *escherichia coli* to biocides: a systems analysis. *Applied and Environmental Microbiology* 86 e00708. 10.1128/AEM.00708-20 32385082 PMC7357472

[B74] Pomposiello P. J. Demple B. (2001). Redox-operated genetic switches: the SoxR and OxyR transcription factors. *Trends Biotechnol.* 19 109–114. 10.1016/S0167-7799(00)01542-0 11179804

[B75] Pontes M. H. Sevostyanova A. Groisman E. A. (2015). When too much ATP is bad for protein synthesis. *J. Mol. Biol.* 427 2586–2594. 10.1016/j.jmb.2015.06.021 26150063 PMC4531837

[B76] Pourret O. (2018). On the necessity of banning the term “heavy metal” from the scientific literature. *Sustainability* 10:2879. 10.3390/su10082879

[B77] Prabhakaran P. Ashraf M. A. Aqma W. S. (2016). Microbial stress response to heavy metals in the environment. *RSC Adv.* 6 109862–109877. 10.1039/c6ra10966g

[B78] Py B. Barras F. (2010). Building Feg-S proteins: bacterial strategies. *Nat. Rev. Microbiol.* 8 436–446. 10.1038/nrmicro2356 20467446

[B79] Quintelas C. Rocha Z. Silva B. Fonseca B. Figueiredo H. Tavares T. (2009). Removal of Cd(II), Cr(VI), Fe(III) and Ni(II) from aqueous solutions by an E. coli biofilm supported on kaolin. *Chem. Eng. J.* 149 319–324. 10.1016/j.cej.2008.11.025

[B80] Ray P. D. Huang B. W. Tsuji Y. (2012). Reactive oxygen species (ROS) homeostasis and redox regulation in cellular signaling. *Cell. Signal.* 24 981–990. 10.1016/j.cellsig.2012.01.008 22286106 PMC3454471

[B81] Robinson M. D. McCarthy D. J. Smyth G. K. (2009). edgeR: a bioconductor package for differential expression analysis of digital gene expression data. *Bioinformatics* 26 139–140. 10.1093/bioinformatics/btp616 19910308 PMC2796818

[B82] Robinson M. D. Oshlack A. (2010). A scaling normalization method for differential expression analysis of RNA-seq data. *Genome Biol.* 11:R25. 10.1186/gb-2010-11-3-r25 20196867 PMC2864565

[B83] Rouch D. A. Lee B. T. O. Morby A. P. (1995). Understanding cellular responses to toxic agents: a model for mechanism-choice in bacterial metal resistance. *J. Ind. Microbiol.* 14 132–141. 10.1007/BF01569895 7766205

[B84] Seo S. W. Kim D. Szubin R. Palsson B. O. (2015). Genome-wide reconstruction of OxyR and SoxRS transcriptional regulatory networks under oxidative stress in *Escherichia coli* K-12 MG1655. *Cell Rep.* 12 1289–1299. 10.1016/j.celrep.2015.07.043 26279566

[B85] Singh K. Singh V. K. (2010). Expression of four methionine sulfoxide reductases in Staphylococcus aureus. *Int. J. Microbiol.* 2012:719594. 10.1155/2012/719594 PMC3261475 22272204

[B86] Skibinski D. A. G. Golby P. Chang Y. S. Sargent F. Hoffman R. Harper R. (2002). Regulation of the hydrogenase-4 operon of *Escherichia coli* by the σ54-dependent transcriptional activators FhlA and HyfR. *J. Bacteriol.* 184 6642–6653. 10.1128/JB.184.23.6642-6653.2002 12426353 PMC135417

[B87] Søballe B. Poole R. K. (1999). Microbial ubiquinones: multiple roles in respiration, gene regulation and oxidative stress management. *Microbiology* 145 1817–1830. 10.1099/13500872-145-8-1817 10463148

[B88] Syversen T. Kaur P. (2012). The toxicology of mercury and its compounds. *J. Trace Elements Med. Biol.* 26 215–226. 10.1016/j.jtemb.2012.02.004 22658719

[B89] Tarver S. Gray D. Loponov K. Das D. B. Sun T. Sotenko M. (2019). Biomineralization of Pd nanoparticles using Phanerochaete chrysosporium as a sustainable approach to turn platinum group metals (PGMs) wastes into catalysts. *Int. Biodeterior. Biodegr.* 143:104724. 10.1016/j.ibiod.2019.104724

[B90] Tatusov R. L. Galperin M. Y. Natale D. A. Koonin E. V. (2000). The COG database: a tool for genome-scale analysis of protein functions and evolution. *Nucleic Acids Res.* 28 33–36. 10.1093/nar/28.1.33 10592175 PMC102395

[B91] The Math Works Inc. (1991). The math works inc. *Simulation* 57:240. 10.1177/003754979105700407

[B92] Tiwari S. Van Tonder A. J. Vilchèze C. Mendes V. Thomas S. E. Malek A. (2018). Arginine-deprivation–induced oxidative damage sterilizes mycobacterium tuberculosis. *Proc. Natl. Acad. Sci. U.S.A.* 115 9779–9784. 10.1073/pnas.1808874115 30143580 PMC6166831

[B93] Trchounian K. Poladyan A. Trchounian A. (2016). Optimizing strategy for *Escherichia coli* growth and hydrogen production during glycerol fermentation in batch culture: effects of some heavy metal ions and their mixtures. *Appl. Energy* 177 335–340. 10.1016/j.apenergy.2016.05.129

[B94] Tyler B. (1978). Regulation of the assimilation of nitrogen compounds. *Annu. Rev. Biochem.* 47 1127–1162. 10.1146/annurev.bi.47.070178.005403 28074

[B95] Vishnukumar P. Vivekanandhan S. Muthuramkumar S. (2017). Plant-mediated biogenic synthesis of palladium nanoparticles: recent trends and emerging opportunities. *ChemBioEng. Rev.* 4 18–36. 10.1002/cben.201600017

[B96] Wackett L. P. Dodge A. G. Ellis L. B. M. (2004). Microbial genomics and the periodic table. *Appl. Environ. Microbiol.* 70 647–655. 10.1128/AEM.70.2.647-655.2004 14766537 PMC348800

[B97] Wang A. Crowley D. E. (2005). Global gene expression responses to cadmium toxicity in *Escherichia coli*. *J. Bacteriol.* 187 3259–3266. 10.1128/JB.187.9.3259-3266.2005 15838054 PMC1082819

[B98] Wang H. Gunsalus R. P. (2000). The nrfA and nirB nitrite reductase operons in *Escherichia coli* are expressed differently in response to nitrate than to nitrite. *J. Bacteriol.* 182 5813–5822. 10.1128/jb.182.20.5813-5822.2000 11004182 PMC94705

[B99] Wang H. Tseng C.-P. Gunsalus R. P. (1999). The napF and narG nitrate reductase operons in *Escherichia coli* are differentially expressed in response to submicromolar concentrations of nitrate but not nitrite. *J. Bacteriol.* 181 5303–5308. 10.1128/jb.181.17.5303-5308.1999 10464201 PMC94036

[B100] Wang S. Deng K. Zaremba S. Deng X. Lin C. Wang Q. (2009). Transcriptomic response of *Escherichia coli* O157:H7 to oxidative stress. *Appl. Environ. Microbiol.* 75 6110–6123. 10.1128/AEM.00914-09 19666735 PMC2753066

[B101] Watanabe S. Kita A. Kobayashi K. Miki K. (2008). Crystal structure of the [2Fe-2S] oxidative-stress sensor SoxR bound to DNA. *Proc. Natl. Acad. Sci. U.S.A.* 105 4121–4126. 10.1073/pnas.0709188105 18334645 PMC2393793

[B102] Weerasinghe J. P. Dong T. Schertzberg M. R. Kirchhof M. G. Sun Y. Schellhorn H. E. (2006). Stationary phase expression of the arginine biosynthetic operon argCBH in *Escherichia coli*. *BMC Microbiol.* 6:14. 10.1186/1471-2180-6-14 16504055 PMC1413537

[B103] Xavier K. B. Bassler B. L. (2005). Regulation of uptake and processing of the quorum-sensing autoinducer AI-2 in *Escherichia coli*. *J. Bacteriol.* 187 238–248. 10.1128/JB.187.1.238-248.2005 15601708 PMC538819

[B104] Xi H. Schneider B. L. Reitzer L. (2000). Purine catabolism in *Escherichia coli* and function of xanthine dehydrogenase in purine salvage. *J. Bacteriol.* 182 5332–5341. 10.1128/JB.182.19.5332-5341.2000 10986234 PMC110974

[B105] Yin C. Zheng L. Zhu J. Chen L. Ma A. (2015). Enhancing stress tolerance by overexpression of a methionine sulfoxide reductase A (MsrA) gene in Pleurotus ostreatus. *Appl. Microbiol. Biotechnol.* 99 3115–3126. 10.1007/s00253-014-6365-4 25573474

[B106] Zereini F. Alt F. (2006). *Palladium Emissions in the Environment.* Berlin: Springer-Verlag.

[B107] Zhang G. Amani M. Chaturvedi A. Tan C. Bullock J. Song X. (2019). Optical and electrical properties of two-dimensional palladium diselenide. *Appl. Phys. Lett.* 114:253102. 10.1063/1.5097825

[B108] Zhang T. Shi X. C. Xia Y. Mai L. Tremblay P. L. (2019). *Escherichia coli* adaptation and response to exposure to heavy atmospheric pollution. *Sci. Rep.* 9:10879. 10.1038/s41598-019-47427-7 31350435 PMC6659633

[B109] Zhang W. Culley D. E. Hogan M. Vitiritti L. Brockman F. J. (2006). Oxidative stress and heat-shock responses in Desulfovibrio vulgaris by genome-wide transcriptomic analysis. *Antonie van Leeuwenhoek* 90 41–55. 10.1007/s10482-006-9059-9 16680520

[B110] Zhang Y. Chen S. Hao X. Su J. Q. Xue X. Yan Y. (2016). Transcriptomic analysis reveals adaptive responses of an *enterobacteriaceae* strain LSJC7 to arsenic exposure. *Front. Microbiol.* 7:636. 10.3389/fmicb.2016.00636 27199962 PMC4852401

[B111] Zhao C. Hartke A. La Sorda M. Posteraro B. Laplace J. M. Auffray Y. (2010). Role of methionine sulfoxide reductases A and B of *Enterococcus faecalis* in oxidative stress and virulence. *Infect. Immun.* 78 3889–3897. 10.1128/IAI.00165-10 20566694 PMC2937430

